# The Predictive Value of Myoglobin for COVID-19-Related Adverse Outcomes: A Systematic Review and Meta-Analysis

**DOI:** 10.3389/fcvm.2021.757799

**Published:** 2021-11-18

**Authors:** Chaoqun Ma, Dingyuan Tu, Jiawei Gu, Qiang Xu, Pan Hou, Hong Wu, Zhifu Guo, Yuan Bai, Xianxian Zhao, Pan Li

**Affiliations:** ^1^Department of Cardiology, Changhai Hospital, Naval Medical University, Shanghai, China; ^2^Department of General Surgery, The Fifth People's Hospital of Shanghai, Fudan University, Shanghai, China

**Keywords:** COVID-19, myoglobin, cardiac troponin I, predictive value, severe illness, mortality

## Abstract

**Objective:** Cardiac injury is detected in numerous patients with coronavirus disease 2019 (COVID-19) and has been demonstrated to be closely related to poor outcomes. However, an optimal cardiac biomarker for predicting COVID-19 prognosis has not been identified.

**Methods:** The PubMed, Web of Science, and Embase databases were searched for published articles between December 1, 2019 and September 8, 2021. Eligible studies that examined the anomalies of different cardiac biomarkers in patients with COVID-19 were included. The prevalence and odds ratios (ORs) were extracted. Summary estimates and the corresponding 95% confidence intervals (95% CIs) were obtained through meta-analyses.

**Results:** A total of 63 studies, with 64,319 patients with COVID-19, were enrolled in this meta-analysis. The prevalence of elevated cardiac troponin I (cTnI) and myoglobin (Mb) in the general population with COVID-19 was 22.9 (19–27%) and 13.5% (10.6–16.4%), respectively. However, the presence of elevated Mb was more common than elevated cTnI in patients with severe COVID-19 [37.7 (23.3–52.1%) vs.30.7% (24.7–37.1%)]. Moreover, compared with cTnI, the elevation of Mb also demonstrated tendency of higher correlation with case-severity rate (Mb, *r* = 13.9 vs. cTnI, *r* = 3.93) and case-fatality rate (Mb, *r* = 15.42 vs. cTnI, *r* = 3.04). Notably, elevated Mb level was also associated with higher odds of severe illness [Mb, OR = 13.75 (10.2–18.54) vs. cTnI, OR = 7.06 (3.94–12.65)] and mortality [Mb, OR = 13.49 (9.3–19.58) vs. cTnI, OR = 7.75 (4.4–13.66)] than cTnI.

**Conclusions:** Patients with COVID-19 and elevated Mb levels are at significantly higher risk of severe disease and mortality. Elevation of Mb may serve as a marker for predicting COVID-19-related adverse outcomes.

**Prospero Registration Number:** https://www.crd.york.ac.uk/prospero/display_record.php?ID=CRD42020175133, CRD42020175133.

## Introduction

Coronavirus disease 2019, caused by severe acute respiratory syndrome coronavirus 2 (SARS-CoV-2), was first reported in Wuhan City, Hubei province of China in December 2019 (1). The pandemic spread rapidly worldwide from China, resulting in 230 million confirmed cases and more than 4 million deaths by September 22, 2021. Clinical manifestations differ greatly among patients with coronavirus disease 2019 (COVID-19), ranging from asymptomatic infections to severe or critical disease and even death (2). Although SARS-CoV-2 was initially thought to be a respiratory tract virus, it has been widely reported that the adverse prognosis of patients with COVID-19 relates largely to the involvement of multisystem organs such as the heart, liver, kidney, brain, and the nervous system (3–5).

Cardiac injury, manifested as the elevation of cardiac biomarkers, namely, cardiac troponin I (cTnI), lactate dehydrogenase (LDH), creatine kinase (CK), CK isomer-MB (CK-MB), myoglobin (Mb), and B-type natriuretic peptide (BNP) or N-terminal pro-B type natriuretic peptide (NT-proBNP), has been detected in numerous patients with COVID-19, and is closely related to the clinical prognosis (6–9). In particular, elevation of cTnI, which was widely reported in several studies, has been identified as an independent variable associated with in-hospital mortality (10).

Nevertheless, elevation of Mb in patients with COVID-19 has been widely mentioned in several studies (11–15). More importantly, Mb presents a potential predictive value in COVID-19-related adverse outcomes. In a study reported by Qin et al., elevated Mb presented with higher frequency on admission and showed the highest overall performance for predicting the risk of COVID-19 mortality among the various cardiac biomarkers (16). However, to the best of our knowledge, a pooled analysis regarding the advantage of Mb in predicting the prognosis of COVID-19 is lacking. Therefore, we conducted a systematic review and meta-analysis to explore the predictive value of elevated Mb for adverse outcomes of patients with COVID-19.

## Methods

### Study Protocol

This study was performed according to the Preferred Reporting Items for Systematic Reviews and Meta-Analyses (PRISMA) statement and Meta-analysis of Observational Studies in Epidemiology (MOOSE) reporting guidelines (17, 18). The protocol was preregistered in the International prospective register of systematic reviews (PROSPERO, CRD42020175133). The detailed definitions of laboratory-confirmed COVID-19 cases and severe illness are described in Supplementary Method S1.

### Search Strategy and Study Selection

Two investigators (DT and JG) independently searched the PubMed, Embase, and Web of Science Core Collection (Clarivate Analytics) databases for relevant articles published between December 2019 and September 8, 2021 using the following keywords: “coronavirus,” “nCoV,” “HCoV,” “SARS-CoV-2,” “COVID^*^,” “NCP^*^,” “cardiac injury,” “cardiac,” “biomarker^*^,” “myocardial,” “heart,” “troponin,” and “myoglobin” alone and in combination. The detailed search strategies are presented in Supplementary Methods S2. After removing duplicate studies, three reviewers (CM, DT, and JG) were assigned to independently screen the titles and abstracts, and then examine the full texts. Any disagreement was resolved by the senior authors (YB and XZ). The inclusion criteria were as follows: (1) diagnosis of COVID-19 according to the World Health Organization interim guidance (19), (2) gives the specific number of COVID-19 patients with the elevation of cTnI and/or Mb, (3) studies in English only, and (4) sample size of ≥10 individuals. The exclusion criteria were as follows: (1) studies with data that could not be reliably extracted, and (2) editorials, comments, expert opinions, case reports.

### Data Extraction and Quality Assessment

Using a predesigned spreadsheet, three authors (DT, CM, and JG) independently extracted the relevant data from the included studies. Corresponding authors were asked via email to clarify or provide additional information. Study quality assessments were performed using the Quality Assessment Forms recommended by the Agency for Healthcare Research and Quality (AHRQ) for cross-sectional studies (Supplementary Methods S3). Studies were defined as high quality if a score of ≥7 was attained. Any conflicts with the assessments were resolved either by consensus or by the adjudicators (XZ and PL).

### Statistical Analysis

Effect estimates were presented as pooled prevalence or odds ratio (OR) with 95% confidence interval (CI) and visualized with forest plots. A fixed or random-effects model was used according to heterogeneity across studies (if *I*^2^ ≤50%, fixed-effects model; if *I*^2^ >50%, random-effects model) (20). We performed Egger's test and the test performed by Peters et al., and visually inspected the funnel plots to investigate publication bias (21). Sensitivity analyses were performed by systematically removing each study in turn to explore its effect on the outcome. All the analyses were performed using R (version 3.5.3), RStudio (version 1.2.1335), and Comprehensive Meta-Analysis.

### Patient and Public Involvement

Patients or the public were not involved in the design, conduct, reporting, and dissemination plans of our research.

## Results

### Literature Search and Study Characteristics

**Figure 1:**
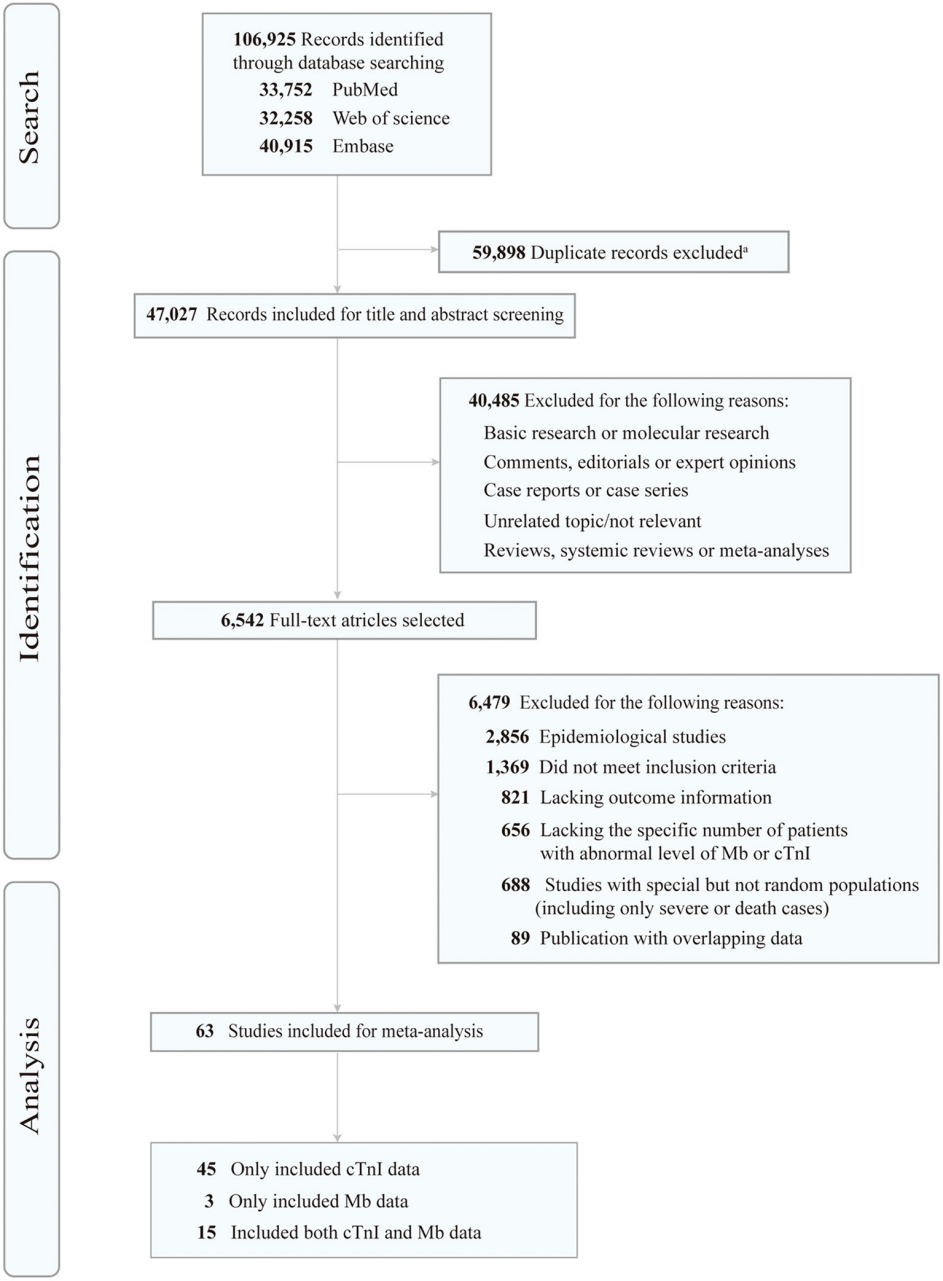
Flow chart of the study selection process. Mb, myoglobin; cTnI, cardiac troponin I. ^a^EndNote software (Clarivate Analytics) was used to remove duplicates.

A total of 106,925 articles were initially retrieved, of which the full texts of 6,542 articles were reviewed (Figure 1). Finally, 63 studies were eligible for our analysis (Table 1 and Supplementary Tables S1, S2), and included 64,319 confirmed patients with COVID-19 who presented to a hospital. All these studies were retrospective observational ones. Of the 63 studies, 31 were conducted in China, 18 in the United States, 5 in Italy, 4 in Spain, 2 in Turkey, and 3 in other countries (Libya, Finland, and Iran) (Supplementary Table S2). Among them, 45 studies only mentioned data of cTnI, 3 studies only mentioned Mb, and 15 studies included both Mb and cTnI. Regarding the differences in Mb or cTnI detection methods and criteria among different hospitals, we listed in Table 1 the average level of Mb or cTnI, cut-off value of abnormal Mb or cTnI, and number of patients with elevated Mb or cTnI in each study. In addition, preexisting cardiovascular conditions, such as the prevalence of coronary artery disease (CAD) and heart failure (HF), and the average level of BNP or NT-proBNP were also summarized (Table 1).

**Table 1:** Characteristics of the included studies.

**Authors**	**No**.	**Cardiovascular condition**	**Mb**	**cTnI**	**Outcome**
Arcari L et al.	111	CAD, 12 (11.0); HF, 8 (7.0)	NA	Average level of cTnI, 17 (5–47) pg/mL; cut-off value, 14 pg/ml; elevated patients, 39/103 (37.9%)	Death
Bardaji' A et al.	186	CAD, 20 (10.8); HF, 14 (7.5)	NA	Elevated patients, 41 (22.0%)	Death, admission to ICU
Bhatla A et al.	700	CAD, 76 (11.0); HF, 88 (13.0); BNP, 2,940 (7,962) pg/mL	NA	Cut-off value, 0.01 ng/mL; elevated patients, 82/373 (22.0%)	NA
Cai Q et al.	298	CAD, 25 (8.4); HF, 7 (2.3)	Average level of Mb, 37.1 (29.2–51.5) μg/L; elevated patients, 10/260 (3.8%)	NA	Death, discharge
Calvo-Fernández A et al.	872	CAD, 59 (6.83); HF, 41 (4.73)	NA	Cut-off value, 14.0 ng/L; elevated patients, 225/651 (34.6%)	Death, admission to ICU, mechanical ventilation
Cao J et al.	102	CAD, 5 (4.9); BNP, 12.2 (0–63.1) pg/mL; NT-pro BNP, 417 (132–1,800) pg/mL	NA	Average level of cTnI, 8.0 (3.0–35.7) pg/mL; cut-off value, 0.026 ng/mL; elevated patients, 15/55 (27.3%)	Discharge, death
Cao J et al.	244	NA	Average level of Mb in severe patients, 39.35 (29.21–74.19) μg/L; Cut-off value, 110 μg/L	Cut-off value, 0.04 ng/mL; elevated patients, 27/244 (11.1%)	Severe COVID-19, death, mechanic ventilation
Cao M et al.	198	CAD, 12 (6.0)	Average level of Mb, 5.9 (2.8–15.7) μg/L; cut-off value, 48.8 μg/L; elevated patients, 33/194 (17.0%)	Average level of cTnI, 0.02 (0.01–0.04) ng/ml; cut-off value, 0.04 ng/mL; elevated patients, 22/194 (11.3%)	Severe COVID-19
Chen N et al.	99	CAD, 40 (40.0)	Average level of Mb, 49.5 (32.2–99.8) μg/L; cut-off value, 146.9 μg/L; elevated patients, 15 (15.2%)	NA	Discharge, death
Chorin E et al.	204	CAD, 25 (12.0); HF, 7 (3.0)	NA	Average level of cTnI, 0.02 (0.01–0.04) ng/Ml; cut-off value, 0.05 ng/mL; elevated patients, 84 (41.2%)	Death
Cipriani A et al.	109	CAD, 18 (17.0); HF, 16 (15.0%); BNP, 90 (22–262) pg/ml	NA	Average level of cTnI, 18.0 (7.0–96.0) ng/L; cut-off value, 32 ng/L for males, 16 ng/L for females; elevated patients, 46 (42.2%)	Death, admission to ICU, discharge
Deng Q et al.	112	CAD, 15 (13.4); HF, 6 (5.4); NT-pro BNP, 430.1 (100.6–2859.3) ng/L	NA	Average level of cTnI, 0.01 (0.00–0.14) ng/ml; cut-off value, 0.04 ng/mL; elevated patients, 42 (37.5%)	Severe COVID-19, death
Elhadi M et al.	1,207	CAD, 25 (2.1)	NA	Cut-off value, 26 pg/mL; elevated patients, 90/292 (30.8%)	Death, admission to ICU
Feng Y et al.	476	CAD, 38 (8.0); BNP, 40.85 (21.64–79.37) pg/ml	Average level of Mb, 18.85 (4.8–51.48) μg/L	Elevated patients, 86/384 (22.4%)	Death, discharge, severe COVID-19
Ferguson J et al.	72	NA	NA	Cut-off value, 0.055 ng/mL; elevated patients, 2/45 (4.4%)	Death, mechanical ventilation, admission to ICU
Ferrante G et al.	332	CAD, 49 (14.5); BNP, 72.5 (34.5–198.0) pg/mL	NA	Average level of cTnI, 11.4 (4.7–37.3) mg/L; cut-off value, 0.02 ng/L; elevated patients, 123 (37.0%)	Death, admission to ICU
Franks C et al.	182	NA	NA	Cut-off value, 0.03 ng/mL; elevated patients, 80/143 (55.9%)	Death
García de Guadiana-Romualdo L et al.	1,280	CAD, 328 (25.6)	NA	Elevated patients, 344 (26.9%)	Death, admission to ICU
Garibaldi BT et al.	832	CAD, 266 (32.0); HF, 127 (15.0); NT-pro BNP 214 (45–960) pg/mL	NA	Elevated patients, 194/682 (28.4%)	Death, severe COVID-19
Guo T et al.	187	CAD, 21 (11.2); NT-pro BNP, 268.4 (75.3–689.1) pg/mL	Average level of Mb, 38.5 (21.0–78.0) μg/L	Elevated patients, 52 (27.8%)	Death
Han H et al.	273	NA	Cut-off value, 110 μg/L; elevated patients, 29/273 (10.6%)	Cut-off value, 0.04 ng/mL; elevated patients, 27/273 (9.9%)	Death, severe COVID-19
Harmouch F et al.	560	Vascular disease, 36 (6.4); HF, 54 (9.6)	NA	Cut-off value, 0.05 ng/mL; elevated patients, 97/482 (20.1%)	Death, mechanical ventilation, admission to ICU
He F et al.	288	CAD, 85 (29.5); BNP, 35 (13–117.5) pg/mL	Elevated patients, 8/276 (2.9%)	Cut-off value, 0.03 ng/mL; elevated patients, 22/190 (11.6%);	Death, admission to ICU
He X et al.	1,031	CAD, 83 (8.1); NT-pro BNP 124 (43–374) pg/mL	NA	Average level of cTnI, 5.3 (2.5–14.0) pg/Ml; elevated patients, 215 (20.9%)	Death
Hu L et al.	323	CAD, 41 (12.7)	NA	Cut-off value, 0.04 pg/mL; elevated patients, 68 (21.1%)	Death, severe COVID-19, mechanical ventilation
Huang C et al.	41	CAD, 6 (15.0)	NA	Average level of cTnI, 3.4 (1.1–9.1) pg/mL; cut-off value, 0.028 ng/mL; elevated patients, 5/41 (12.2%)	Death, severe COVID-19, discharge
Huang J et al.	98	CAD, 6 (6.0); BNP 119 (54–392) pg/mL	NA	Cut-off value, 0.0229 ng/Ml; elevated patients, 7 (7.1%)	Death, discharge, severe COVID-19
Huang R et al.	202	CAD, 5 (2.5)	NA	Elevated patients, 2/103 (1.9%)	Admission to ICU, mechanical ventilation, severe COVID-19
Karbalai Saleh S et al.	386	CAD, 97 (25.1)	NA	Cut-off value, 26 ng/L for males, 11 ng/L for females; elevated patients, 115 (29.8%)	Death, admission to ICU
Lala A et al.	2,736	CAD, 453 (16.6); HF, 276 (10.1)	NA	Cut-off value, 0.03 ng/mL; OR for in-hospital mortality, 1.75 (1.37–2.24); elevated patients, 985 (36.0%)	Death
Li C et al.	2,068	CAD, 182 (8.8); HF, 14 (0.7); NT-pro BNP 108 (36–370) pg/mL	Average level of Mb, 40.7 (28.4–73.8) μg/L; elevated patients, 174/1,554 (11.2%)	Average level of cTnI, 4.2 (1.9–11.0) pg/mL; elevated patients, 181 (8.8%)	Death, severe COVID-19
Li X et al.	548	CAD, 34 (6.2)	NA	Cut-off value, 15.6 pg/mL; elevated patients, 119 (21.7%)	Discharge, death, severe COVID-19
Maeda T et al.	181	CAD, 36 (19.9); HF, 24/180 (13.3)	NA	Elevated patients, 54 (29.8%)	Death
Majure D et al.	6,247	CAD, 833 (13.0); HF, 529 (9.0)	NA	Cut-off value, 0.045 ng/mL; elevated patients, 1,821 (29.1%)	Death, admission to ICU, mechanical ventilation
Manocha KK et al.	446	CAD, 94 (21.1); HF, 38 (8.5); BNP 84 (25–300) pg/mL	NA	Average level of cTnI, 0.05 (0–0.34) ng/Ml; cut-off value, 0.34 ng/mL; elevated patients, 112 (25.1%)	Death, admission to ICU
Merugu GP et al.	217	NA	NA	Elevated patients, 34/201 (16.9%)	Death
Mikami T et al.	6,493	NA	NA	Average level of cTnI, 0.03 (0.02–0.10) ng/dl; cut-off value, 0.03 ng/dL; elevated patients, 1,312/2,526 (51.9%)	Death
Özyilmaz S et al.	105	CAD, 14 (21.1)	NA	Average level of cTnI, 2.6 (0–1774.5) pg/mL^a^; cut-off value, 7.8 ng/mL; elevated patients, 21 (20.0%)	Death
Palaiodimos L et al.	200	CAD, 33 (16.5); HF, 34 (17.0)	NA	Cut-off value, 0.01 ng/mL; elevated patients, 56 (28.0%)	Mortality, intubation, O_2_ requirement, ARDS, ICU, AKI, RRT, length of stay
Peiró ÓM et al.	196	CAD, 19 (9.7); HF, 14 (7.1)	NA	Average level of cTnI, 14 (4–37) ng/L; cut-off value, 21 ng/L; elevated patients, 77 (39.3%)	Death, admission to ICU, mechanical ventilation
Price-Haywood E et al.	3,481	CAD, 139 (4.0); HF, 128 (3.7)	NA	Cut-off value, 0.06 ng/mL; elevated patients, 270/1,084 (24.9%)	Death, admission to ICU
Qin J et al.	3,219	CAD, 206 (6.4)	Elevated patients, 228/1,895 (12.0%); HR for in-hospital mortality, 6.84 (4.95–9.45) AUC for mortality, 0.83 (0.80–0.86)	Elevated patients, 95/1,462 (6.5%); HR for in-hospital mortality, 9.59 (6.36–14.47); AUC for in-hospital mortality, 0.78 (0.73–0.84)	Death
Richardson S et al.	5,700	CAD, 595 (11.1); HF, 371 (6.9); BNP, 385.5 (160–1996.8), *n* = 1,818	NA	Elevated patients, 801/3,533 (22.7%)	Admission to ICU, mechanical ventilation, kidney replacement therapy, Death
Schiavone M et al.	674	HF, 111 (16.5)	NA	Average level of cTnI, 18 (8–40) ng/L; elevated patients, 130 (19.3%)	Death, admission to ICU, mechanical ventilation
Shah P et al.	309	CAD, 28 (9.1); HF, 65 (21.0)	NA	Elevated patients, 116 (37.5%)	Death, admission to ICU, mechanical ventilation
Shen Y et al.	325	NA	Cut-off value, 48.8 μg/L; elevated patients, 28/325 (8.6%)	Cut-off value, 0.04 ng/mL; elevated patients, 80/325 (24.6%)	Death, discharge
Singh N et al.	276	Vascular disease, 49 (17.8); HF, 56 (20.3)	NA	Cut-off value, 0.017 ng/mL; elevated patients, 132/276 (47.8%) OR for in-hospital mortality, 4.43 (1.61–12.19)	Death
Stefanini G et al.	397	Prior MI, 33/395 (8.4); HF, 18/395 (4.6); BNP, 67 (30–191) pg/mL	NA	Average level of cTnI, max 10.8 (4.3–39.5) ng/L, baseline 7.8 (4.5–25.6) ng/L; elevated patients, 130 (32.7%)	Death, admission to ICU, discharge
Suleyman G et al.	463	CAD, 59 (12.7); HF, 49 (10.6)	NA	Elevated patients, 107 (23.1%)	Death, admission to ICU
Tanboga IH et al.	14,855	CAD, 2,341 (15.3); HF, 776 (5.1)	NA	Average level of cTnI, 0.08 (0.00–0.28) ng/mL; elevated patients, 1,027 (6.9%)	Death, admission to ICU, mechanical ventilation
Tomasoni D et al.	692	CAD, 148 (21.4); HF, 90 (13.0); NT-pro BNP 303 (96–1,201) pg/mL	NA	Elevated patients, 272/605 (45.0%)	Death
Wang D et al.	138	CAD, 20 (14.5)	NA	Average level of cTnI, 6.4 (2.8–18.5) pg/mL; cut-off value, 0.0262 ng/mL; Elevated patients, 10 (7.2%)	Admission to ICU
Wang Z et al.	293	CAD, 21 (7.2)	Average level of Mb, 57.6 (30.8–116.4) μg/L; cut-off value, 110 μg/L; elevated patients, 58/213 (27.2%)	Average level of cTnI, 0.007 (0.006–0.046) ng/mL; cut-off value, 0.0796 ng/mL; elevated patients, 36/216 (16.7%)	Death
Wei J et al.	101	CAD, 5 (5.0); NT-pro BNP, 71.2 (31.6–237.5) pg/mL	NA	Average level of cTnI, 6.8 (4.3–10.1) pg/mL; cut-off value, 0.014 ng/mL; elevated patients, 16 (15.8%)	Death, severe case, admission to ICU, mechanical ventilation
Wu Y et al.	125	CAD, 11 (8.8); BNP, 65.0 (23.0–178.0) pg/mL	Average level of Mb, 35.0 (27.7–75.65) μg/L; cut-off value, 154.9 μg/L; elevated patients, 14 (11.2%)	Average level of cTnI, 3.9 (1.9–10.3) pg/ml; cut-off value, 0.0342 ng/mL; elevated patients, 10 (8.0%)	Long-term hospitalization
Xu P et al.	703	CAD, 35 (5.0)	Elevated patients, 33/181 (18.2%)	NA	Death, admission to ICU, mechanical ventilation
Zeng J et al.	416	CAD, 13 (3.1); HF, 5/57 (8.8)	Cut-off value, 100 μg/L; elevated patients, 30/174 (17.2%)	Cut-off value, 0.026 ng/mL; elevated patients, 29/345 (8.4%)	Death, discharge
Zhang G et al.	221	CAD, 22 (10.0)	NA	Average level of cTnI, 7.6 (3.6–21.5) pg/mL; cut-off value, 0.0262 ng/mL; elevated patients, 17 (7.7%)	Discharge, death, severe COVID-19
Zhang Q et al.	41	CAD, 1 (2.4)	Average level of Mb, 26.0 (19.7–118.6) μg/L; elevated patients, 11 (26.8%)	Average level of cTnI; 1.5 (0.8–5.0) ng/mL; elevated patients, 41 (100%)	Severe COVID-19
Zhang Y et al.	166	CAD, 30 (18.1); NT-proBNP, 179.0 (67.0–457.0) pg/mL	Average level of Mb, 54.8 (33.8–127.2) μg/L; cut-off value, 106 μg/L; elevated patients, 28/166 (16.9%)	Average level of cTnI, 5.0 (2.2–10.7) pg/mL; cut-off value, 0.0156 ng/mL; elevated patients, 17 /166 (10.2%)	Discharge, death
Zhao M et al.	1,000	CAD, 60 (6.0)	Average level of Mb, 44.54 (28.5–85.05) μg/L; cut-off value, 110 μg/L; elevated patients, 132/754 (17.5%)	Average level of cTnI, 0.006 (0.006–0.018) ng/mL; cut-off value, 0.0796 ng/mL; elevated patients, 66/758 (8.7%)	Death, discharge
Zhao X et al.	91	HF, 14 (15.4)	NA	Cut-off value, 0.01 ng/mL; elevated patients, 3/88 (3.4%)	Death, discharge
Zhou F et al.	191	CAD, 15 (8.0); HF, 44 (23.0)	NA	Average level of cTnI, 4.1 (2.0–14.1) ng/mL; cut-off value, 28 ng/mL; elevated patients, 24/145 (16.6%)	Death, admission to ICU

*No., confirmed number of patients with coronavirus disease 2019(COVID-19); Mb, myoglobin; cTnI, cardiac troponin I; CAD, coronary artery disease; HF, heart failure; BNP, B-type natriuretic peptide; NT-proBNP, N-terminal pro-B type natriuretic peptide; ICU, intensive-care unit; NA, data not available*.

a *Median (range)*.

### Incidence of cTnI/Mb Elevation

**Figure 2:**
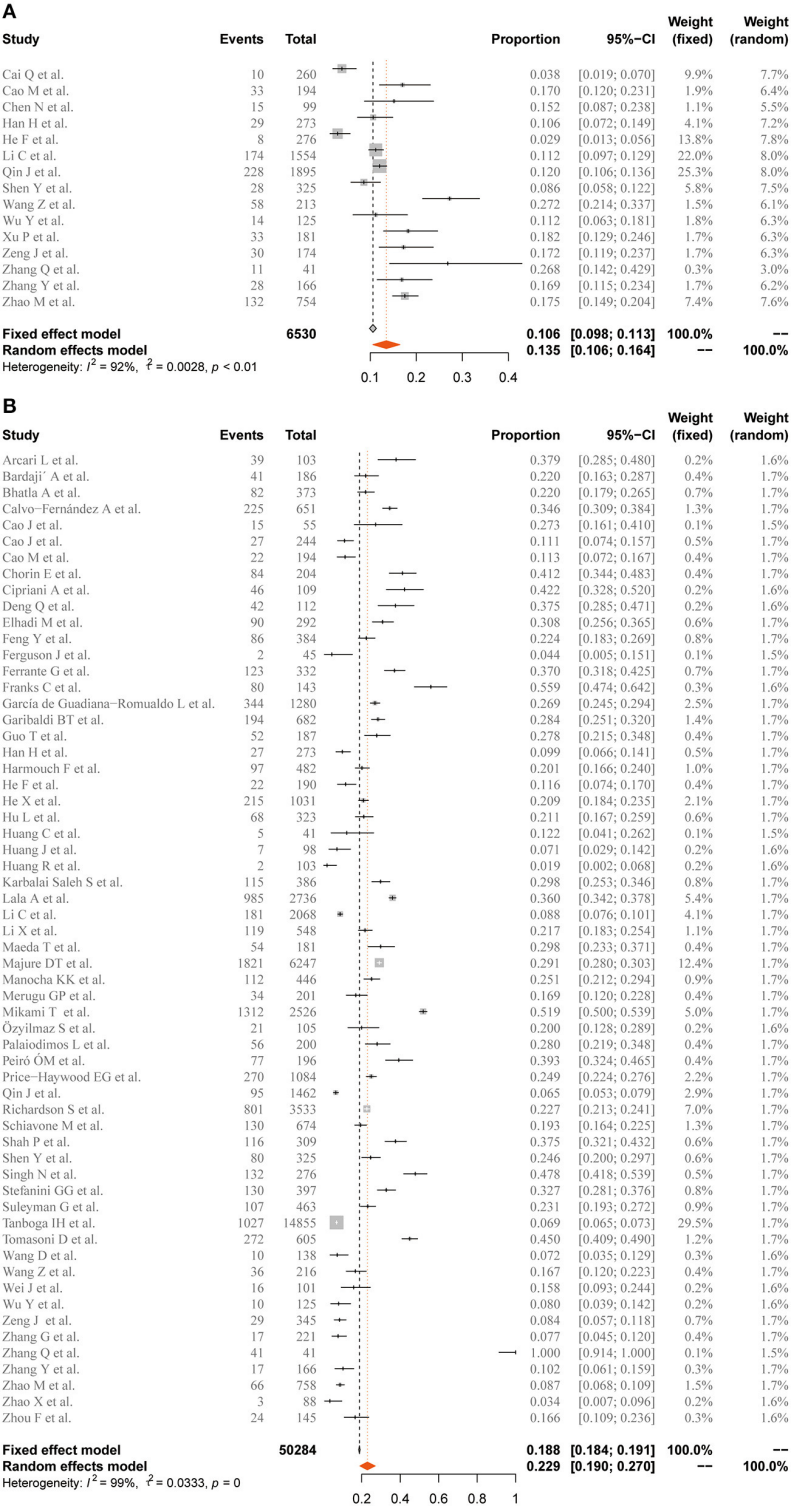
Forest plot for the pooled prevalence of elevated **(A)** Mb and **(B)** cTnI in general population. Mb, myoglobin; cTnI, cardiac troponin I. Proportions are presented with fixed-effects when *I*^2^ ≤ 50% and random-effects otherwise.

**Figure 3:**
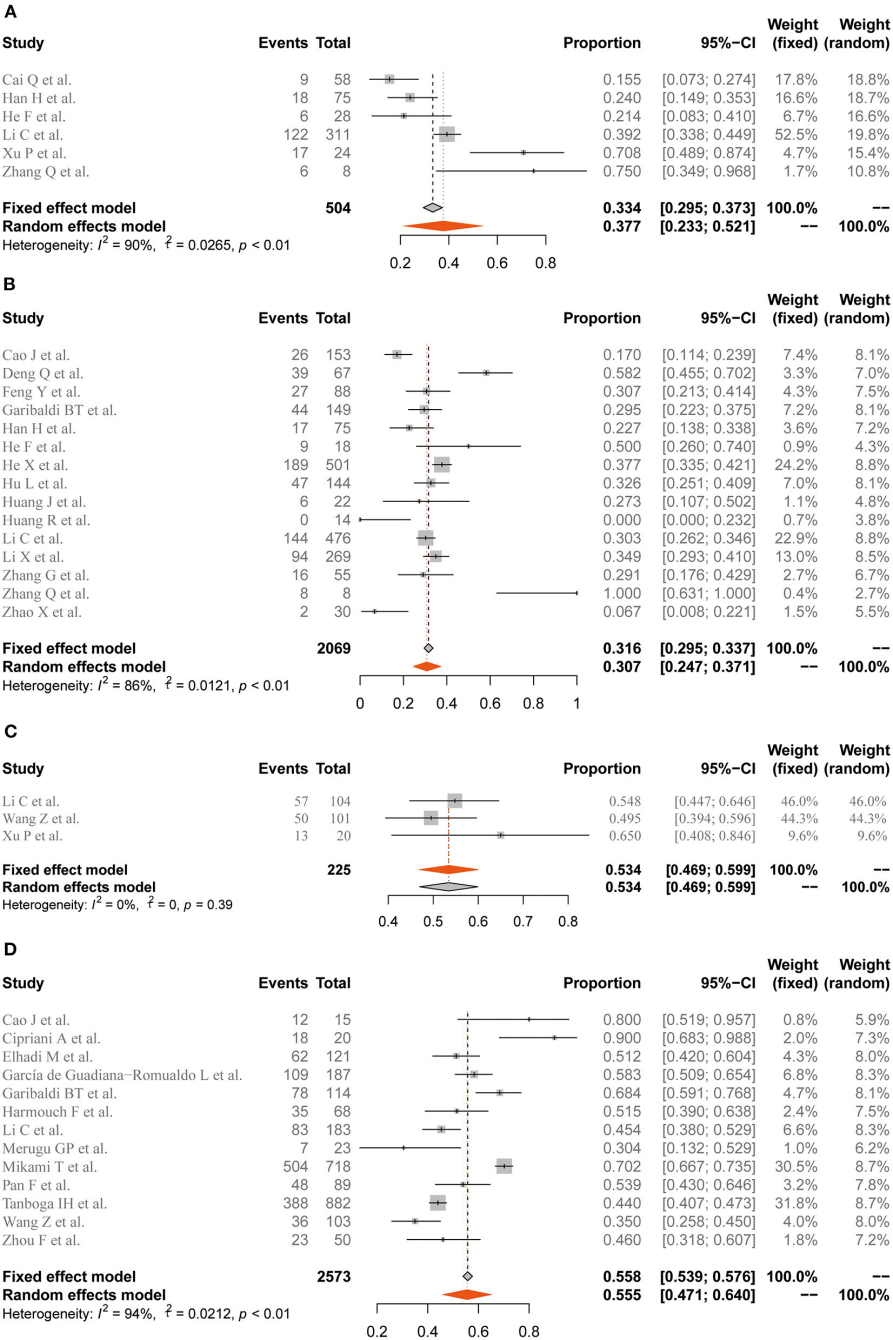
Forest plot for the pooled prevalence of elevated Mb and cTnI in the severe disease and non-survivor groups. **(A)** Prevalence of elevated Mb in the severe disease group. **(B)** Prevalence of elevated cTnI in the severe disease group. **(C)** Prevalence of elevated Mb in the non-survivor group. **(D)** Prevalence of elevated cTnI in the non-survivor group. Mb, myoglobin; cTnI, cardiac troponin I. Proportions are presented with fixed-effects when *I*^2^ ≤50% and random-effects otherwise.

Among the 63 included studies, the pooled case-severity rate (CSR), case-fatality rate (CFR), and intensive-care unit (ICU)-admission rate were 31.3 (95% CI 23.2–39.4%, *I*^2^ = 99%), 12.5 (95% CI 10.7–14.6%, *I*^2^ = 98%), and 20.1% (95% CI 15.3–24.9%, *I*^2^ = 99%) (Supplementary Figure S1). The prevalence of elevated cTnI and Mb in the general population with COVID-19 was 22.9 (95% CI 19–27%, *I*^2^ = 99%) and 13.5% (95% CI 10.6–16.4%, *I*^2^ = 92%), respectively (Figure 2). Furthermore, the meta-analysis showed that elevated cTnI occurred in 30.7% (24.7–37.1%, *I*^2^ = 86%) of the patients in the severe disease group, while the estimated rate of elevated Mb was 37.7% (23.3–52.1%, *I*^2^ = 90%) in patients with severe COVID-19. For the non-survivor group, the elevation rate of Mb and cTnI was 53.4 (95% CI 46.9–59.9%, *I*^2^ = 0%) and 55.5% (95% CI 47.1–64%, *I*^2^ = 94%), respectively (Figure 3).

**Figure 4:**
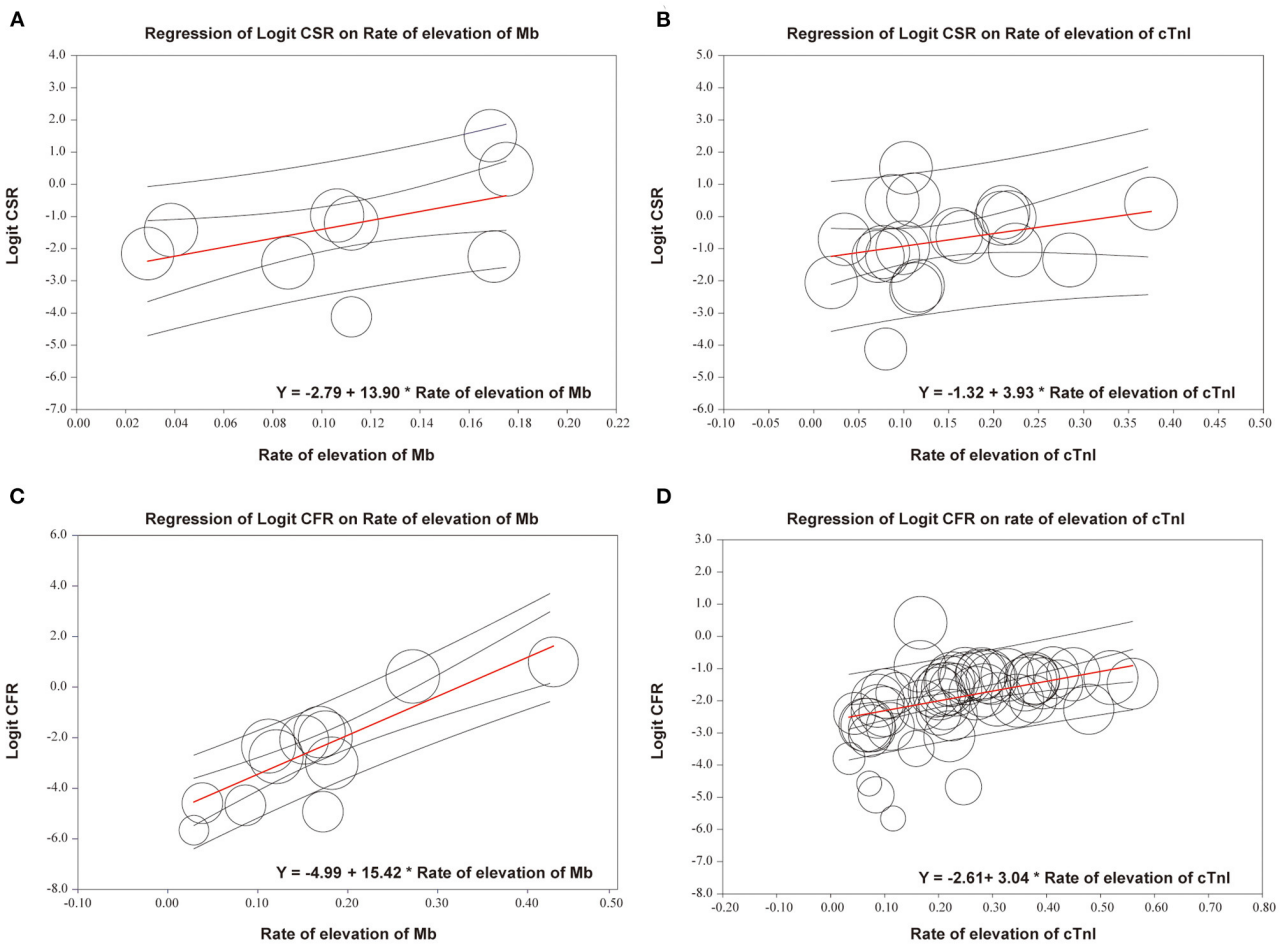
Meta-regression of logit CSR or CFR on the rate of elevation of Mb or cTnI. **(A)** Regression of logit CSR on rate of elevation of Mb; *R* = 13.9, 95% CI 3.51–24.29, *p* < 0.01. **(B)** Regression of logit CSR on rate of elevation of cTnI; *r* = 3.93, 95% CI 0–8.52, *p* < 0.05. **(C)** Regression of logit CFR on rate of elevation of Mb; *r* = 15.42, 95% CI 11.2–19.65, *p* < 0.0001. **(D)** Regression of logit CFR on rate of elevation of cTnI; *r* = 3.04, 95% CI 1.84–4.25, *p* < 0.0001. CSR, case-severity rate; CFR, case-fatality rate; Mb, myoglobin; cTnI, cardiac troponin I. Each circle represents one study; size of the circle is proportional to the population size of each study.

Meta-regression demonstrated that both CSR and CFR were positively associated with the proportion of patients with elevated cTnI or Mb. Regarding logit CSR, the prevalence of elevated Mb showed tendency of higher regression coefficient compared with cTnI (Mb: *r* = 13.9, [95% CI 3.51–24.29, *p* < 0.01] vs. cTnI: *r* = 3.93, [95% CI 0–8.52, *p* < 0.05]). A similar trend was observed in logit CFR (Mb: *r* = 15.42, [95% CI 11.2–19.65, *p* < 0.0001] vs. cTnI: *r* = 3.04, [95% CI 1.84–4.25, *p* < 0.0001]) (Figure 4).

### Risk of Elevated cTnI/Mb for Adverse Outcomes

**Figure 5:**
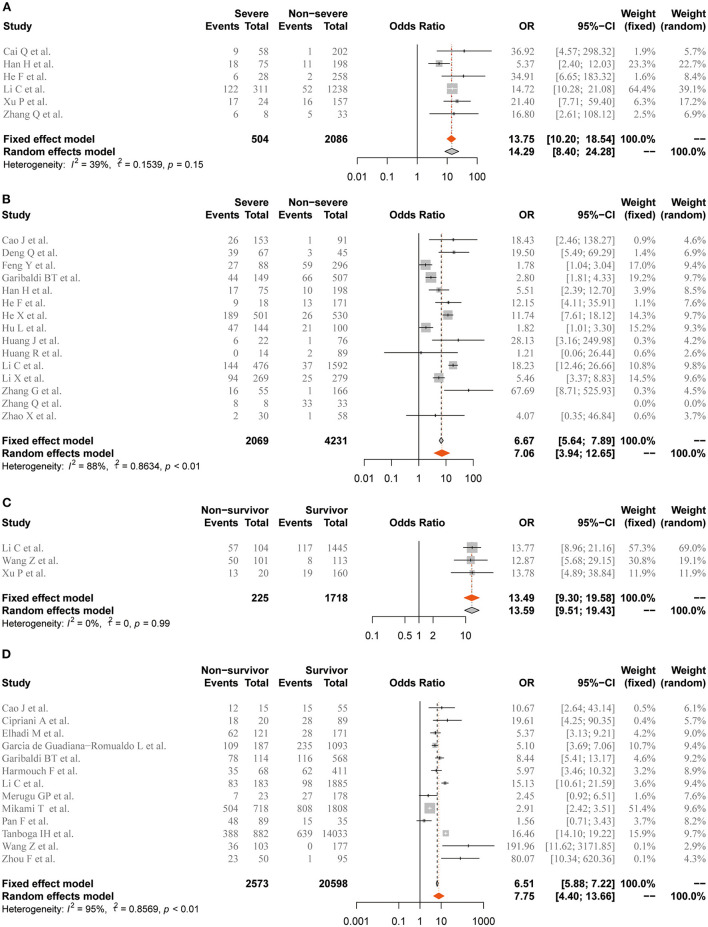
Forest plot for the association of coronavirus disease 2019 (COVID-19)-related adverse outcomes with abnormal level of Mb or cTnI. **(A)** Severe illness and elevation of Mb. **(B)** Severe illness and elevation of cTnI. **(C)** In-hospital mortality and elevation of Mb. **(D)** In-hospital mortality and elevation of cTnI. Mb, myoglobin; cTnI, cardiac troponin I. Odds ratios (ORs) are presented with fixed-effects when *I*^2^ ≤50% and random-effects otherwise.

The ORs of elevation of Mb/cTnI for the development of severe illness and death were further estimated. In the overall analysis, patients COVID-19 and elevated cTnI were at higher risk of severe illness (OR = 7.06, 95% CI 3.94–12.65, n = 15, *I*^2^ = 88%). Nevertheless, elevated Mb showed tendency of better predictive value for severe illness (OR = 13.75, 95% CI 10.2–18.54, *n* = 6, *I*^2^ = 39%) compared with cTnI. Regarding in-hospital mortality, elevated cTnI (OR = 7.75, 95% CI 4.4–13.66, *n* = 13, *I*^2^ = 95%) and Mb (OR = 13.49, 95% CI 9.3–19.58, *n* = 3, *I*^2^ = 0%) were associated with COVID-19-related deaths (Figure 5).

### Sensitivity Analysis and Publication Bias

Sequential removal of each trial from the analysis revealed no meaningful differences (Supplementary Figure S2). We observed no evidence of publication bias by inspecting the funnel plot or with Egger's test, Begger's test or the test used by Peters et al. (*p* > 0.05; Supplementary Figure S3).

## Discussion

This systematic review and meta-analysis of 63 high-quality retrospective studies systematically investigated the predictive value of Mb for COVID-19-related severe disease or death compared with cTnI. The main findings of the study are as follows: (1) more patients with COVID-19-related severe disease showed elevated Mb compared with elevated cTnI; (2) elevated Mb presented obvious superiority over cTnI for predicting severe illness, showing 3-fold higher meta-regression coefficient and 2-fold higher OR; (3) furthermore, Mb elevation was more strongly associated with high risk of COVID-19-related death compared with cTnI.

Severe acute respiratory syndrome coronavirus 2 has been reported to be more contagious than previously discovered human coronaviruses (22), with the progression of the COVID-19 pandemic worldwide, there has been increasing concern regarding the “destructive power” of SARS-CoV-2 for multiple system organ damage, such as in the heart, liver, kidney, brain, and the nervous system (5, 23). Among them, myocardial injury is an important manifestation (6). Madjid et al. reported that up to 15% of hospitalized patients with COVID-19 exhibit myocardial injury, with some developing significant cardiac complications, such as biventricular heart failure, arrhythmias, and cardiogenic shock (9, 24). Liu et al. demonstrated that the mortality rate of patients with COVID-19 and cardiovascular disease was as high as 10.5%, which was 11.67 times higher than that of patients with COVID-19 with no preexisting conditions (25). Consistently, our analysis showed that the pooled incidence rate of cardiac injury was 22.9% in the general population, while the rate increased to 55.5% in the non-survivor group, indicating that cardiac injury was common in patients with COVID-19, especially those with poor prognosis.

Abnormal levels of cardiac biomarkers, including cTnI, CK-MB, Mb, and NT-proBNP, have been identified as indicators for COVID-19-related poor prognosis, such as severe illness (26), ICU admission and in-hospital mortality (27, 28). However, there is no consensus on the optimal biomarker for predicting COVID-19-related outcomes. cTnI elevation has been widely studied for its high prevalence in patients with COVID-19. However, in a study by Qin et al., elevated Mb presented with obviously higher frequency on admission compared with cTnI (12 vs. 6.5%) (16). Similarly, our subgroup analysis revealed that elevated Mb was more common in patients with severe COVID-19 than cTnI. Several recent studies have highlighted elevated cTnI as an important risk factor for adverse outcomes, such severe illness (29, 30), ICU admission (31, 32), and death (10, 26, 33). However, our meta-regression analysis suggested that the elevation rate of Mb presented 3-fold stronger association with CSR and 5-fold stronger association with CFR than cTnI. Notably, elevated Mb level showed higher risk of severe illness and mortality compared with cTnI. The results suggested that Mb may serve as a better biomarker for the severity of COVID-19. Accordingly, the dynamic monitoring of Mb might facilitate timely initiation of intensive care, thereby reducing the risk of other adverse events, such as COVID-19-related death.

Myoglobin is an iron and oxygen-binding protein that plays an important role in the storage of oxygen in skeletal and cardiac muscles (34). Previously, it was generally believed that Mb, while sensitive, was not specific for cardiac injury *per se*. Therefore, the prognostic value of Mb as a marker of myocardial injury in patients with COVID-19 has not been taken seriously (35). However, our meta-analysis suggested that Mb has a potential advantage over cTnI in predicting COVID-19-related adverse outcomes, such as the occurrence of severe illness and death. The mechanistic link between Mb and COVID-19 prognosis is unclear, but it may be the distribution of Mb in skeletal muscle besides myocardium, making it more sensitive to the dynamics of systemic states (36). de Andrade-Junior et al. reported that patients with severe COVID-19 are prone to develop muscle wasting and impaired muscle function (37). Moreover, Mb can be rapidly released into the blood in response to inflammatory stimuli (38). Wang et al. reported that oxidized Mb can act as a useful marker of myocardial inflammation (39). Furthermore, emerging evidence suggests that inflammatory responses, such as lymphopenia and cytokine storm, are closely associated with severe COVID-19 and high mortality (40, 41). Therefore, besides myocardial injury, the link between elevated Mb and COVID-19 prognosis may also be explained by inflammation and muscle injury. In addition to SARS-CoV-2 infection, increased Mb may also be caused by other preexisting comorbidities, such as chronic obstructive pulmonary disease (COPD), liver diseases, kidney diseases, and cardiovascular diseases, which have also been identified as risk factors for COVID-19 severity and mortality (42–45). Taken together, elevated Mb may be involved in damage directly caused by SARS-COV-2 infection and subsequent multiple organ failure, which partly explains the predictive value of Mb for adverse prognosis of COVID-19.

In the past year, the development and application of vaccines against SARS-CoV-2 brought hope to people worldwide. Notably, for the prevention of adverse outcomes of COVID-19, Chung et al. reported that two doses of mRNA COVID-19 vaccines were highly effective against symptomatic infection and severe consequences (46). Cornberg et al. demonstrated that priority vaccination for COVID-19 in patients with chronic liver diseases may be an important measure to intervene in the course of severe COVID-19 (47). However, the exact efficacy of COVID-19 vaccines against various comorbidities associated with myoglobin elevation is unknown and remains to be elucidated.

This meta-analysis had several potential limitations. First, all the studies included in this meta-analysis were retrospective, and there were relatively few studies involving both Mb and cTnI. Hence, the superiority of Mb over cTnI in predicting value should be interpreted as an observational conclusion. Further high-quality comparative studies are needed to confirm the difference between Mb and cTnI in predicting prognosis of COVID-19. Second, because of the nature of meta-regression and high heterogeneity across the analyses, we were unable to obtain a definite causal relationship between elevated Mb and poor prognosis of COVID-19. The potential sources of heterogeneity include different cutoffs of elevated cTnI or Mb, mean ages (48, 49), and sex ratios (50) in different studies. Therefore, considering the confounding factors, our results need to be further confirmed by rigorous prospective studies and randomized controlled trials. Third, because of the limited number of included studies, this meta-analysis did not analyze the predictive value of CK-MB, NT-proBNP, LDH, and other cardiac markers except Mb and cTnI. Fourth, studies enrolled in this meta-analysis had a relatively short follow-up period. Therefore, the predictive value of Mb for long-term prognosis of COVID-19 needs to be further explored.

In summary, this meta-analysis showed that patients with COVID-19 and elevated Mb levels are at higher risk of severe disease and mortality. Hence, elevated Mb could be used as a predictor of adverse outcomes in COVID-19. However, high-quality studies are required to confirm these findings and establish the link between elevated Mb and prognosis of patients with COVID-19.

## Data Availability Statement

The original contributions presented in the study are included in the article/Supplementary Material, further inquiries can be directed to the corresponding author/s.

## Author Contributions

PL, XZ, and YB were the judicators and contributed to the conception of the study. CM, DT, JG, YB, ZG, and HW designed the protocol. CM, DT, and JG searched the databases and finished data extraction, quality assessment, and statistical analysis. CM and DT wrote the first draft of the manuscript. All authors reviewed the manuscript, provided critical revision, and have approved the final version for publication.

## Funding

This study was supported in part by grants from the Chinese Natural Science Foundation (81870356 to PL), Shanghai Rising-Star Program (20QA1409000 to PL), and 234 Discipline Promotion Foundation of Changhai (2020YXK010 to YB).

## Conflict of Interest

The authors declare that the research was conducted in the absence of any commercial or financial relationships that could be construed as a potential conflict of interest.

## Publisher's Note

All claims expressed in this article are solely those of the authors and do not necessarily represent those of their affiliated organizations, or those of the publisher, the editors and the reviewers. Any product that may be evaluated in this article, or claim that may be made by its manufacturer, is not guaranteed or endorsed by the publisher.
