# Supplementary material for: The Predictive Value of Myoglobin for COVID-19-Related Adverse Outcomes: A Systematic Review and Meta-Analysis
Source: Front Cardiovasc Med. 2021 Nov 18;8:757799. doi: 10.3389/fcvm.2021.757799 (PMC8636904; doi:10.3389/fcvm.2021.757799)
Supplement: Supplementary file 1 [file Data_Sheet_1.PDF]

Electronic supplementary material for

The predictive value of myoglobin for COVID-19-related adverse outcomes: a systematic review and meta-analysis

Methods S1. Definition

Methods S2. Search strategies

Methods S3. Quality assessment

Table S1. Quality assessment form

Table S2. Characteristics of the included studies

Figure S1. Forest plot of the overall prevalence of severe illness (A) and in-hospital mortality (B) and ICU-admission (C) of COVID-19 patients

Figure S2. Forest plot showing result of sensitivity analysis after excluding each study in turn

Figure S3. Funnel plot

References

Abbreviations: Mb, myoglobin; cTnI, cardiac troponin I; ICU, intensive care unit**.**

Methods S1. Definition

1. Laboratory-confirmed case

Laboratory-confirmed case was defined as the presence of SARS-CoV-2 in respiratory specimens (including nasal and pharyngeal swabs) detected by the reverse-transcriptase polymerase chain reaction (RT-PCR), which was conducted in accordance with the protocol established by the World Health Organization.[1] Practice of the diagnostic criteria was based on the recommendation by the National Institute for Viral Disease Control and Prevention of CDC (Available at http://ivdc.chinacdc.cn/kyjz/202001/t20200121_211337.html).

2. Severe disease

Severe disease was defined as meeting one of the following criteria: 1) presence of shortness of breath with a respiratory rate ≥ 30 breaths/minute; 2) an oxygen saturation (SpO2) ≤ 93% in the resting state; 3) hypoxemia defined as an arterial

partial pressure of oxygen divided by the fraction of inspired oxygen (PaO2/FiO2 ratio) ≤ 300 mmHg; 4) presence of radiographic progression, defined as ≥ 50% increase of target lesion within 24-48 hours. The criterion was based on the Chinese COVID-19 prevention and control program (6th edition, http://www.nhc.gov.cn/yzygj/s7653p/202002/8334a8326dd94d329df351d7da8aefc2.shtml, accessed Feb 18, 2020) and American Thoracic Society guideline[2].

Methods S2. Search strategies

Search strategy for PubMed (September 8, 2021)

#	Searches	Results
#1	((covid-19[Title/Abstract] OR 2019 novel coronavirus[Title/Abstract] OR covid19[Title/Abstract] OR sars-cov-2[Title/Abstract] OR 2019-ncov[Title/Abstract] OR coronavirus disease 2019[Title/Abstract] OR coronavirus disease-19[Title/Abstract] OR Wuhan virus*[Title/Abstract] OR severe acute respiratory syndrome coronavirus 2[Title/Abstract] OR wuhan coronavirus[Title/Abstract] OR sars2[Title/Abstract] OR wuhan seafood market pneumonia virus*[Title/Abstract])) AND (("2019/12/01"[Date - Publication] : "2021/09/08"[Date - Publication]))	167,289
#2	("Coronavirus"[Mesh]) AND (("2019/12/01"[Date - Publication]: "2021/09/08"[Date - Publication]))	83,604
#3	#1 OR #2	171,191
#4	((laboratory[Title/Abstract] OR biomarker*[Title/Abstract] OR marker*[Title/Abstract] OR blood[Title/Abstract] OR serum[Title/Abstract] OR myocardial[All fields] OR heart[All fields] OR cardiac[All fields] OR cardio*[All fields] OR troponin[All fields] OR cTnI[All fields] OR myoglobin[All fields] OR Mb[All fields])) AND (("2019/12/01"[Date - Publication] : "2021/09/08"[Date - Publication]))	653,900

#5	#3 AND #4	34,606
#6	5# Filters: English	33,752

Search strategy for Web of Science Core Collection (September 8, 2021)

#	Searches	Results
#1	TS=((((((((((((covid-19) OR (2019 novel coronavirus)) OR (covid19)) OR (sars-cov-2)) OR (2019-ncov)) OR (coronavirus disease 2019)) OR (coronavirus disease-19)) OR (Wuhan virus*)) OR (severe acute respiratory syndrome coronavirus 2)) OR (wuhan coronavirus)) OR (sars2)) OR (wuhan seafood market pneumonia virus*)) *Indexes=SCI-EXPANDED, SSCI, A&HCI, CPCI-S, CPCI-SSH, ESCI, CCR-EXPANDED, IC Timespan=All years*	184,871
#2	TS=((((laboratory OR biomarker*) OR marker*) OR blood) OR serum) *Indexes=SCI-EXPANDED, SSCI, A&HCI, CPCI-S, CPCI-SSH, ESCI, CCR-EXPANDED, IC Timespan=All years*	4,882,468
#3	All=(((((((myocardial OR heart) OR cardiac) OR cardio*) OR troponin) OR cTnI) OR myoglobin) OR Mb) *Indexes=SCI-EXPANDED, SSCI, A&HCI, CPCI-S, CPCI-SSH, ESCI, CCR-EXPANDED, IC Timespan=All years*	3,993,968
#4	#3 OR #2 *Indexes=SCI-EXPANDED, SSCI, A&HCI, CPCI-S, CPCI-SSH, ESCI, CCR-EXPANDED, IC Timespan=All years*	8,052,433
#5	#4 AND #1 *Indexes=SCI-EXPANDED, SSCI, A&HCI, CPCI-S, CPCI-SSH, ESCI, CCR-EXPANDED, IC Timespan=All years*	33,412
#6	(#4 AND #1) AND LANGUAGE: (English) *Indexes=SCI-EXPANDED, SSCI, A&HCI, CPCI-S, CPCI-SSH, ESCI, CCR-EXPANDED, IC Timespan=2019-2021*	32,258

Search strategy for Embase (September 8, 2021)

#	Searches	Results
#1	laboratory:ti,ab,kw OR biomarker*:ti,ab,kw OR marker*:ti,ab,kw OR blood:ti,ab,kw OR serum:ti,ab,kw	5,438,834
#2	myocardial OR heart OR cardiac OR cardio* OR troponin OR ctni OR myoglobin OR mb	4,611,584
#3	'covid 19':ti,ab,kw OR '2019 novel coronavirus':ti,ab,kw OR covid19:ti,ab,kw OR 'sars cov 2':ti,ab,kw OR '2019 ncov':ti,ab,kw OR 'coronavirus disease 2019':ti,ab,kw OR 'coronavirus disease-19':ti,ab,kw OR 'wuhan virus*':ti,ab,kw OR 'severe acute respiratory syndrome coronavirus 2':ti,ab,kw OR 'wuhan coronavirus':ti,ab,kw OR sars2:ti,ab,kw OR 'wuhan seafood market pneumonia virus*':ti,ab,kw	171,120
#4	#1 OR #2	8,888,780
#5	#3 AND #4	42,185
#6	#3 AND #4 AND [english]/lim AND [1-12-2019]/sd NOT [8-9-2021]/sd	40,915

Methods S3. Quality assessment

Quality Assessment Forms recommended by Agency for Healthcare Research and Quality (AHRQ) for cross-sectional study

1) Define the source of information (survey, record review)
2) List inclusion and exclusion criteria for exposed and unexposed subjects (cases and controls) or refer to previous publications
3) Indicate time period used for identifying patients
4) Indicate whether or not subjects were consecutive if not population-based
5) Indicate if evaluators of subjective components of study were masked to other aspects of the status of the participants
6) Describe any assessments undertaken for quality assurance purposes (eg.

test/retest of primary outcome measurements)

7) Explain any patient exclusions from analysis
8) Describe how confounding was assessed and/or controlled.
9) If applicable, explain how missing data were handled in the analysis
10) Summarize patient response rates and completeness of data collection
11) Clarify what follow-up, if any, was expected and the percentage of patients for which incomplete data or follow-up was obtained

The methodological quality of the studies included was assessed using an 11-item checklist which was recommended by Agency for Healthcare Research and Quality (AHRQ). An item would be scored '0' if it was answered 'NO' or 'UNCLEAR'; if it was answered 'YES', then the item scored '1'. Article quality was assessed as follows: low quality = 0-3; moderate quality = 4-7; high quality = 8-11.

Table S1. Quality assessment form

Study	A	B	C	D	E	F	G	H	I	J	K	Quality score
Arcari L et al.	1	1	1	1	1	1	1	0	1	1	1	10
Bardajı´ A et al.	1	1	1	1	0	0	1	1	1	0	1	8
Bhatla A et al.	1	0	1	1	1	0	1	1	1	1	1	9
Cai Q et al.	1	1	1	1	1	1	1	1	1	1	1	11
Calvo-Fernández A et al.	1	1	1	1	0	1	1	1	1	1	0	9
Cao J et al.	1	0	1	1	1	0	1	1	1	1	1	9
Cao J et al.	1	1	1	1	1	1	1	1	1	1	1	11
Cao M et al.	1	0	1	1	1	0	1	0	1	1	1	8
Chen N et al.	1	1	1	1	1	1	1	1	1	1	1	11
Chorin E et al.	1	1	0	1	1	1	1	1	1	1	1	10
Cipriani A et al.	1	1	1	1	1	1	1	1	1	1	0	10
Deng Q et al.	1	0	1	1	1	0	1	0	1	1	0	7
Elhadi M et al.	1	1	1	1	1	1	1	1	1	1	0	10

Feng Y et al.	1	1	1	1	1	1	1	0	1	1	1	10
Ferguson J et al.	1	1	1	1	1	1	1	1	1	1	1	11
Ferrante G et al.	1	1	1	1	1	1	1	1	1	1	1	11
Franks C et al.	1	1	1	1	1	1	1	1	1	1	1	11
García de Guadiana-Romualdo L et al.	1	1	1	1	0	1	1	1	1	1	0	9
Garibaldi BT et al.	1	1	1	1	1	1	1	1	1	1	1	11
Guo T et al.	1	1	1	1	1	1	1	1	1	1	1	11
Han H et al.	1	0	1	1	1	0	1	1	1	1	0	8
Harmouch F et al.	1	1	1	1	1	1	1	1	1	1	1	11
He F et al.	1	1	1	1	1	1	1	1	1	1	1	11
He X et al.	1	1	1	1	1	1	1	1	1	1	0	10
Hu L et al.	1	1	1	1	1	1	1	1	1	1	1	11
Huang C et al.	1	1	1	1	1	1	1	1	1	1	1	11
Huang J et al.	1	1	1	1	0	0	1	1	1	0	1	8
Huang R et al.	1	1	1	1	1	1	1	1	1	1	1	11
Karbalai Saleh S et al.	1	0	1	1	1	0	1	1	1	1	1	9
Lala A et al.	1	1	1	1	1	1	1	0	1	1	1	10
Li C et al.	1	1	1	1	1	0	1	1	1	1	1	10
Li X et al.	1	0	1	1	1	0	1	1	1	1	1	9
Maeda T et al.	1	1	1	1	0	1	1	1	1	1	0	9
Majure D et al.	1	0	1	1	1	0	1	0	1	1	0	7
Manocha KK et al.	1	1	1	1	1	1	1	1	1	1	0	10
Merugu GP et al.	1	1	1	1	0	0	1	1	1	0	1	8
Mikami T et al.	1	1	1	1	1	1	1	0	1	1	1	10
Özyılmaz S et al.	1	1	1	1	0	0	1	1	1	0	1	8
Palaiodimos L et al.	1	1	1	1	1	1	1	1	1	1	1	11

Peiró ÓM et al.	1	1	1	1	1	1	1	1	1	1	0	10
Price-Haywood E et al.	1	1	1	1	1	1	1	1	1	1	1	11
Qin J et al.	1	1	1	1	1	1	1	1	1	1	1	11
Richardson S et al.	1	1	1	1	1	1	1	1	1	1	1	11
Schiavone M et al.	1	1	1	1	1	0	1	1	1	1	1	10
Shah P et al.	1	1	1	1	0	0	1	1	1	0	1	8
Shen Y et al.	1	0	1	1	1	1	1	1	1	1	1	10
Singh N et al.	1	1	1	1	1	1	1	1	1	1	1	11
Stefanini G et al.	1	0	1	1	1	0	1	1	1	1	1	9
Suleyman G et al.	1	1	1	1	1	1	1	0	1	1	1	10
Tanboğa IH et al.	1	1	1	1	0	1	1	1	1	1	0	9
Tomasoni D et al.	1	1	1	1	1	1	1	1	1	1	0	10
Wang D et al.	1	0	1	1	1	0	1	1	1	1	0	8
Wang Z et al.	1	0	1	1	1	0	1	0	1	1	1	8
Wei J et al.	1	1	1	1	1	1	1	1	1	1	1	11
Wu Y et al.	1	1	1	1	1	1	1	1	1	1	1	11
Xu P et al.	1	0	1	1	1	0	1	1	1	1	1	9
Zeng J et al.	1	1	0	1	1	1	1	0	1	1	1	9
Zhang G et al.	1	0	1	1	1	1	1	0	1	1	0	8
Zhang Q et al.	1	1	1	1	1	1	1	1	1	1	0	10
Zhang Y et al.	1	0	1	1	1	1	1	1	1	1	1	10
Zhao M et al.	1	0	1	1	1	0	1	0	1	1	1	8
Zhao X et al.	1	0	1	1	1	1	1	0	1	1	1	9
Zhou F et al.	1	0	1	1	1	1	1	1	1	1	1	10

Table S2 Characteristics of the included studies

Author	Study period	Country (city)	No.	Median age (IQR)	Male n (%)	CSR n (%)	CFR n (%)	Cardiovascular condition	Mb	cTnI	Outcome	Quality Score [a]
Arcari L et al.[3]	2020.03.15 - 2020.04.30	Rome, Italy	111	72.0 (17.0) [b]	51 (46.0)	NA	23 (21.0)	CAD, 12 (11.0); HF, 8 (7.0)	NA	Average level of cTnI,17 (5-47) pg/mL; cut-off value, 14 pg/ml; elevated patients, 39/103 (37.9%)	Death	10
Bardajı´ A et al.[4]	2020.03.16-2020.04.16	Spain	186	67.5 (52.5-77.5)	111 (59.7)	NA	37 (19.9)	CAD, 20 (10.8); HF, 14 (7.5)	NA	Elevated patients, 41 (22.0%)	Death, admission to ICU	8
Bhatla A et al.[5]	2020.03.06 - 2020.03.19	Pennsylvania, the U.S.	700	50.0 (18.0) [b]	314 (45.0)	NA	30 (4.3)	CAD, 76 (11.0); HF, 88 (13.0); BNP, 2940 (7962) pg/mL	NA	Cut-off value, 0.01 ng/mL; elevated patients, 82/373 (22.0%)	NA	9
Cai Q et al.[6]	2020.01.11 - 2020.03.06	Shenzhen, China	298	47.5 (33.0-61.0)	145 (48.66)	58 (19.5)	3 (1.0)	CAD, 25 (8.4); HF, 7 (2.3)	Average level of Mb, 37.1 (29.2-51.5) ug/L; elevated patients, 10/260 (3.8%)	NA	Death, discharge	11
Calvo-Fernández A et al.[7]	2020.02.27-2020.05.01	Barcelona, Spain	872	62.3 (18.1) [b]	486 (55.7)	NA	104 (11.9)	CAD, 59 (6.83); HF, 41 (4.73)	NA	Cut-off value, 14.0 ng/L; elevated patients, 225/651 (34.6%)	Death, admission to ICU mechanical ventilation	9
Cao J et al.[8]	2020.01.03 - 2020.02.15	Wuhan, China	102	54.0 (37.0-67.0)	53 (52.0)	NA	17 (16.7)	CAD, 5 (4.9); BNP, 12.2 (0-63.1) pg/mL, NT-pro BNP, 417 (132-1800) pg/mL	NA	Average level of cTnI,8.0 (3.0-35.7) pg/mL; cut-off value, 0.026 ng/mL； elevated patients, 15/55 (27.3%)	Discharge, death	9
Cao J et al.[9]	2020.02.06 - 2020.02.21	Wuhan, China	244	62.6 (13.4) [b]	133 (54.5)	153 (62.7)	14 (5.74)	NA	Average level of Mb in severe patients, 39.35 (29.21-74.19) ug/L; Cut-off value, 110 ug/L	Cut-off value, 0.04 ng/mL； elevated patients, 27/244 (11.1%)	Severe COVID-19, death, mechanic ventilation	11
Cao M et al.[10]	2020.01.20 - 2020.02.15	Shanghai, China	198	50.1 (16.3) [b]	101 (51.0)	19 (9.6)	NA	CAD, 12 (6.0)	Average level of Mb,5.9 (2.8-15.7) ug/L; cut-off value, 48.8 ug/L； elevated patients, 33/194 (17.0%)	Average level of cTnI,0.02 (0.01-0.04) ng/ml; cut-off value, 0.04 ng/mL； elevated patients, 22/194 (11.3%)	Severe COVID-19	8
Chen N et al.[11]	2020.01.01 - 2020.01.20	Wuhan, China	99	55.0 (13.1) [b]	67 (68.0)	NA	11 (11.0)	CAD, 40 (40.0)	Average level of Mb,49.5 (32.2-99.8) ug/L; cut-off value, 146.9 ug/L; elevated patients, 15 (15.2%)	NA	Discharge, death	11
Chorin E et al.[12]	NA	New York, the U.S.	204	64.0 (13.0) [b]	156 (76.0)	NA	50 (23.0)	CAD, 25 (12.0); HF, 7 (3.0)	NA	Average level of cTnI, 0.02 (0.01-0.04) ng/Ml; cut-off value, 0.05 ng/mL; elevated patients, 84 (41.2%)	Death	10
Cipriani A et al.[13]	2020.02.26 - 2020.03.31	Italy	109	71.0 (60.0-81.0)	73 (67.0)	NA	20 (18.3)	CAD, 18 (17.0); HF, 16 (15.0%); BNP, 90 (22-262) pg/ml	NA	Average level of cTnI,18.0 (7.0-96.0) ng/L; cut-off value, 32 ng/L for males,16 ng/L for females; elevated patients, 46 (42.2%)	Death, admission to ICU, discharge	10
Deng Q et al.[14]	2020.01.06 - 2020.02.20	Wuhan, China	112	65.0 (49.0-70.8)	57 (59.0)	67 (59.8)	14 (12.5)	CAD, 15 (13.4); HF, 6 (5.4); NT-pro BNP, 430.1 (100.6-2859.3) ng/L	NA	Average level of cTnI, 0.01 (0.00-0.14) ng/ml; cut-off value, 0.04 ng/mL； elevated patients, 42 (37.5%)	Severe COVID-19, death	7

Elhadi M et al.[15]	2020.03.24-2020.12.03	Libya	1207	56 (40-64)	811 (67.2)	NA	149 (12.3)	CAD, 25 (2.1)	NA	Cut-off value, 26 pg/mL; elevated patients, 90/292 (30.8%)	Death, admission to ICU	10
Feng Y et al.[16]	2020.01.01 - 2020.02.25	3 provinces, China	476	53.0 (40.0-64.0)	271 (56.9)	124 (26.5)	38 (8.0)	CAD, 38 (8.0); BNP, 40.85 (21.64-79.37) pg/ml	Average level of Mb,18.85 (4.8-51.48) ug/L	Elevated patients, 86/384 (22.4%)	Death, discharge, severe COVID-19	10
Ferguson J et al.[17]	2020.03.13 - 2020.05.02	Standford, the U.S.	72	NA	NA	NA	6 (8.3)	NA	NA	Cut-off value, 0.055 ng/mL; elevated patients, 2/45 (4.4%)	Death, mechanical ventilation, admission to ICU	11
Ferrante G et al.[18]	2020.02.25 - 2020.04.02	Helsinki, Finland	332	66.9 (55.4-75.5)	237 (71.4)	NA	68 (20.5)	CAD, 49 (14.5); BNP, 72.5 (34.5-198.0) pg/mL	NA	Average level of cTnI, 11.4 (4.7-37.3) mg/L; cut-off value, 0.02 ng/L; elevated patients, 123 (37.0%)	Death, admission to ICU	11
Franks C et al.[19]	NA	Washington, the U.S.	182	64.0 (19.0-98.0)	103 (56.6)	NA	34 (18.7)	NA	NA	Cut-off value, 0.03 ng/mL; elevated patients, 80/143 (55.9%)	Death	11
García de Guadiana-Romualdo L et al.[20]	2020.03.01-2020.05.12	Spain	1280	67 (55-77)	750 (58.6)	NA	187 (14.6)	CAD, 328 (25.6)	NA	Elevated patients, 344 (26.9%)	Death, admission to ICU	9
Garibaldi BT et al.[21]	2020.03.04-2020.06.27	Washington, the U.S.	832	63 (49-75)	443 (53.2)	171 (20.6)	131 (15.7)	CAD, 266 (32.0); HF, 127 (15.0); NT-pro BNP 214 (45-960) pg/mL	NA	Elevated patients, 194/682 (28.4%)	Death, severe COVID-19	11
Guo T et al.[22]	2020.01.23 - 2020.02.25	Wuhan, China	187	58.5 (14.7) [b]	91 (48.7)	NA	43 (23.0)	CAD, 21 (11.2); NT-pro BNP, 268.4 (75.3-689.1) pg/mL	Average level of Mb,38.5 (21.0-78.0) ug/L	Elevated patients, 52 (27.8%)	Death	11
Han H et al.[23]	2020.01.01 - 2020.02.18	Wuhan, China	273	NA	97 (35.5)	75 (27.5)	24 (8.79)	NA	Cut-off value, 110 ug/L; elevated patients, 29/273 (10.6%)	Cut-off value, 0.04 ng/mL; elevated patients, 27/273 (9.9%)	Death, severe COVID-19	8
Harmouch F et al.[24]	2020.03.01 - 2020.04.15	Pennsylvania, the U.S.	560	63.0 (24.0) [b]	319 (57.1)	NA	81 (14.4)	Vascular disease, 36 (6.4); HF, 54 (9.6)	NA	Cut-off value, 0.05 ng/mL; elevated patients, 97/482 (20.1%)	Death, mechanical ventilation, admission to ICU	11
He F et al.[25]	2020.01.15 - 2020.03.10	Guangzhou, China	288	48.5 (34.3-62.0)	131 (45.5)	30 (10.4)	1 (0.3)	CAD, 85 (29.5); BNP, 35 (13-117.5) pg/mL	Elevated patients, 8/276 (2.9%)	Cut-off value, 0.03 ng/mL; elevated patients, 22/190 (11.6%);	Death, admission to ICU	11
He X et al.[26]	2020.02.01-2020.03.29	Wuhan, China	1031	63 (52-70)	538 (52.2)	501 (48.6)	165 (16.0)	CAD, 83 (8.1); NT-pro BNP 124 (43-374) pg/mL	NA	Average level of cTnI, 5.3 (2.5-14.0) pg/Ml; elevated patients, 215 (20.9%)	Death	10
Hu L et al.[27]	2020.01.08 - 2020.03.10	Wuhan, China	323	61 (23-91) [c]	166 (51.4)	172 (53.3)	35 (10.8)	CAD, 41 (12.7)	NA	Cut-off value, 0.04 pg/mL; elevated patients, 68 (21.1%)	Death, severe COVID-19, mechanical ventilation	11
Huang C et al.[28]	2019.12 - 2020.01.02	Wuhan, China	41	49.0 (41.0-58.0)	30 (73.0)	NA	6 (15.0)	CAD, 6 (15.0)	NA	Average level of cTnI,3.4 (1.1-9.1) pg/mL; cut-off value, 0.028 ng/mL； elevated patients, 5/41 (12.2%)	Death, severe COVID-19, discharge	11
Huang J et al.[29]	2020.01.17-2020.02.16	Guangzhou, China	98	44.0 (33.0-62.3)	46 (47.0)	22 (22.4)	1 (1.0)	CAD, 6 (6.0); BNP 119 (54-392) pg/mL	NA	Cut-off value, 0.0229 ng/Ml; elevated patients, 7 (7.1%)	Death, discharge, severe COVID-19	8

Huang R et al.[30]	2020.01.22 - 2020.02.10	Jiangsu, China	202	44.0 (33.0-54.0)	116 (57.4)	23 (11.4)	0	CAD, 5 (2.5)	NA	Elevated patients, 2/103 (1.9%)	Admission to ICU, mechanical ventilation, severe COVID-19	11
Karbalai Saleh S et al.[31]	2020.03 - 2020.05	South Tehran, Iran	386	59.5 (15.8) [b]	236 (61.1)	NA	77 (19.9)	CAD, 97 (25.1)	NA	cut-off value, 26 ng/L for males,11 ng/L for females; elevated patients, 115 (29.8%)	Death, admission to ICU	9
Lala A et al.[32]	2020.02.27 - 2020.04.12	New York, the U.S.	2736	66.4 (15.8)	1630 (59.6)	NA	506 (18.5)	CAD, 453 (16.6); HF, 276 (10.1)	NA	Cut-off value, 0.03 ng/mL; OR for in-hospital mortality,1.75 (1.37-2.24); elevated patients, 985 (36.0%)	Death	10
Li C et al.[33]	2020.01.29 - 2020.04.01	Wuhan, China	2068	63.0 (51.0-70.0)	1005 (48.6)	476 (23.0)	183 (8.8)	CAD, 182 (8.8); HF, 14 (0.7); NT-pro BNP 108 (36-370) pg/mL	Average level of Mb,40.7 (28.4-73.8) ug/L; elevated patients, 174/1554 (11.2%)	Average level of cTnI,4.2 (1.9-11.0) pg/mL; elevated patients, 181 (8.8%)	Death, severe COVID-19	10
Li X et al.[34]	2020.01.26 - 2020.03.03	Wuhan, China	548	60.0 (48.0-69.0)	279 (50.9)	269 (49.1)	90/545 (16.5)	CAD, 34 (6.2)	NA	Cut-off value, 15.6 pg/mL; elevated patients, 119 (21.7%)	Discharge, death, severe COVID-19	9
Maeda T et al.[35]	2020.03.13-2020.03.31	New York, the U.S.	181	64.0 (16.6) [b]	101 (55.8)	NA	38/178 (21.3)	CAD, 36 (19.9); HF, 24/180 (13.3)	NA	Elevated patients, 54 (29.8%)	Death	9
Majure D et al.[36]	2020.03.01 - 2020.04.27	New York, the U.S.	6247	66.0 (56.0-77.0)	3740 (60.0)	NA	1397 (22.0)	CAD, 833 (13.0); HF, 529 (9.0)	NA	Cut-off value, 0.045 ng/mL； elevated patients, 1821 (29.1%)	Death, admission to ICU, mechanical ventilation	7
Manocha KK et al.[37]	2020.03.03-2020.04.06	New York, the U.S.	446	65.0 (15.2) [b]	291 (65.3)	NA	95 (21.3)	CAD, 94 (21.1); HF, 38 (8.5) BNP 84 (25-300) pg/mL	NA	Average level of cTnI, 0.05 (0-0.34) ng/Ml; cut-off value, 0.34 ng/mL; elevated patients, 112 (25.1%)	Death, admission to ICU	10
Merugu GP et al. [38]	2020.03.25-2020.06.16	Northwest Ohio, the U.S.	217	63.1 (17.8) [b]	102 (47.0)	NA	23 (10.6)	NA	NA	Elevated patients, 34/201 (16.9%)	Death	8
Mikami T et al.[39]	2020.03.12 - 2020.04.17	New York, the U.S.	6493	59.0 (43.0-72.0)	3538 (54.5)	NA	858 (13.2)	NA	NA	Average level of cTnI,0.03 (0.02-0.10) ng/dl; cut-off value, 0.03 ng/dL； elevated patients,1312/2526 (51.9%)	Death	10
Özyılmaz S et al.[40]	2020.03.20-2020.06.20	Istanbul, Turkey	105	45 (20-87) [c]	76 (72.4)	NA	9 (8.6)	CAD, 14 (21.1)	NA	Average level of cTnI, 2.6 (0-1774.5) pg/mL [c]; cut-off value, 7.8 ng/mL elevated patients, 21 (20.0%)	Death	8
Palaiodimos L et al.[41]	2020.03.09 - 2020.04.12	New York, the U.S.	200	64.0 (50.0-73.5)	98 (49.0)	NA	48 (24.0)	CAD, 33 (16.5); HF, 34 (17.0)	NA	Cut-off value, 0.01 ng/mL; elevated patients, 56 (28.0%)	Mortality, intubation, O2 requirement, ARDS, ICU, AKI, RRT, length of stay	11
Peiró ÓM et al.[42]	2020.03.16-2020.05.15	Tarragona, Spain	196	67.5 (53.5-78.0)	117 (59.7)	NA	37 (18.9)	CAD, 19 (9.7); HF, 14 (7.1)	NA	Average level of cTnI, 14 (4-37) ng/L; cut-off value, 21 ng/L elevated patients, 77 (39.3%)	Death, admission to ICU mechanical ventilation	10
Price-Haywood E et al.[43]	2020.03.01 - 2020.04.11	Louisiana, the U.S.	3481	54.0	1394 (40.0)	NA	326/1382 (23.6)	CAD, 139 (4.0); HF, 128 (3.7)	NA	Cut-off value, 0.06 ng/mL； elevated patients, 270/1084 (24.9%)	Death, admission to ICU	11

Qin J et al. [44]	2019.12.31 - 2020.03.04	Hubei, China	3219	57.0 (45.0-66.0)	1535 (47.7)	NA	193 (6.0)	CAD, 206 (6.4)	Elevated patients, 228/1895 (12.0%); HR for in-hospital mortality,6.84 (4.95-9.45) AUC for mortality,0.83 (0.80-0.86)	Elevated patients, 95/1462 (6.5%); HR for in-hospital mortality,9.59 (6.36-14.47); AUC for in-hospital mortality,0.78 (0.73-0.84)	Death	11
Richardson S et al.[45]	2020.03.01 - 2020.04.04	New York, the U.S.	5700	63.0 (52.0-75.0)	3437 (60.3)	NA	553/2634 (21.0)	CAD, 595 (11.1); HF, 371 (6.9); BNP, 385.5 (160-1996.8), n=1818	NA	Elevated patients,801/3533 (22.7%)	Admission to ICU, mechanical ventilation, kidney replacement therapy, Death	11
Schiavone M et al.[46]	2020.02.23 - 2020.05.01	Italy	674	60.8 (15.9) [b]	406 (60.2)	NA	105 (15.6)	HF, 111 (16.5)	NA	Average level of cTnI,18 (8-40) ng/L; elevated patients, 130 (19.3%)	Death, admission to ICU, mechanical ventilation	10
Shah P et al.[47]	2020.03.02-2020.06.07	Georgia, the U.S.	309	NA	178 (57.3)	NA	66 (21.4)	CAD, 28 (9.1); HF, 65 (21.0)	NA	Elevated patients, 116 (37.5%)	Death, admission to ICU mechanical ventilation	8
Shen Y et al.[48]	2020.01.20 - 2020.02.29	Shanghai, China	325	51.0 (31.0-64.0)	168 (51.7)	26 (8.0)	3 (0.92)	NA	Cut-off value, 48.8 ug/L; elevated patients, 28/325 (8.6%)	Cut-off value, 0.04 ng/mL; elevated patients, 80/325 (24.6%)	Death, discharge	10
Singh N et al.[49]	2020.03.23 - 2020.04.13	Chicago, the U.S.	276	62.0 (50.0-73.0)	130 (47.1)	NA	26 (9.4)	Vascular disease, 49 (17.8); HF, 56 (20.3)	NA	Cut-off value, 0.017 ng/mL; elevated patients, 132/276 (47.8%) OR for in-hospital mortality, 4.43 (1.61-12.19)	Death	11
Stefanini G et al.[50]	2019.12 - 2020.04.01	Italy	397	66.7 (55.0-75.7)	267 (67.3)	NA	92 (23.2)	Prior MI, 33/395 (8.4); HF, 18/395 (4.6); BNP, 67 (30-191) pg/mL	NA	Average level of cTnI, max 10.8 (4.3-39.5) ng/L, baseline 7.8 (4.5-25.6) ng/L; elevated patients, 130 (32.7%)	Death, admission to ICU, discharge	9
Suleyman G et al.[51]	2020.03.09 - 2020.03.27	Michigan, the U.S.	463	57.5 (16.8)	204 (44.1)	NA	16 (3.5)	CAD, 59 (12.7); HF, 49 (10.6)	NA	Elevated patients, 107 (23.1%)	Death, admmission to ICU	10
Tanboğa IH et al.[52]	2020.03.11-2020.06.22	Turkey	14855	49 (36-62)	8272 (54.0)	NA	882 (5.9)	CAD, 2341 (15.3); HF, 776 (5.1)	NA	Average level of cTnI 0.08 (0.00-0.28) ng/mL; elevated patients, 1027 (6.9%)	Death, admission to ICU mechanical ventilation	9
Tomasoni D et al.[53]	2020.03.01-2020.04.09	Italy	692	67.4 (13.2) [b]	481 (69.5)	NA	163 (23.6)	CAD, 148 (21.4) HF, 90 (13.0) NT-pro BNP 303 (96-1201) pg/mL	NA	Elevated patients, 272/605 (45.0%)	Death	10
Wang D et al.[54]	2020.01.01 - 2020.02.03	Wuhan, China	138	56.0 (42.0-68.0)	75 (54.3)	NA	6 (4.3)	CAD, 20 (14.5)	NA	Average level of cTnI,6.4 (2.8-18.5) pg/mL; cut-off value, 0.0262 ng/mL; Elevated patients, 10 (7.2%)	Admission to ICU	8
Wang Z et al.[55]	2019.12.30 - 2020.02.29	Wuhan, China	293	59.2 (42.8-73.1)	138 (47.1)	NA	NA	CAD, 21 (7.2)	Average level of Mb,57.6 (30.8-116.4) ug/L; cut-off value, 110 ug/L; elevated patients, 58/213 (27.2%)	Average level of cTnI,0.007 (0.006-0.046) ng/mL; cut-off value, 0.0796 ng/mL; elevated patients, 36/216 (16.7%)	Death	8
Wei J et al.[56]	2020.01.16 - 2020.03.10	Sichuan, China	101	49.0 (34.0-62.0)	54 (53.5)	37 (36.6)	3 (3.0)	CAD, 5 (5.0); NT-pro BNP, 71.2 (31.6-237.5) pg/mL	NA	Average level of cTnI,6.8 (4.3-10.1) pg/mL; cut-off value, 0.014 ng/mL; elevated patients, 16 (15.8%)	Death, severe case, admission to ICU, mechanical ventilation	11

Wu Y et al.[57]	2020.02.10 - 2020.03.30	Wuhan, China	125	55.0 (40.0-68.5)	66 (52.8)	2 (1.6)	0	CAD, 11 (8.8); BNP, 65.0 (23.0-178.0) pg/mL	Average level of Mb,35.0 (27.7-75.65) ug/L; cut-off value, 154.9 ug/L； elevated patients, 14 (11.2%)	Average level of cTnI,3.9 (1.9-10.3) pg/ml; cut-off value, 0.0342 ng/mL； elevated patients, 10 (8.0%)	Long-term hospitalization	11
Xu P et al.[58]	2020.01.10 - 2020.03.13	8 proviences, China	703	46.1 (15.2) [b]	382 (54.3)	55 (7.8)	33 (4.7)	CAD, 35 (5.0)	Elevated patients, 33/181 (18.2%)	NA	Death, admission to ICU, mechanical ventilation	9
Zeng J et al.[59]	2020.01.11 - 2020.04.01	Shenzhen, China	416	NA	198 (47.6)	NA	3 (0.7)	CAD, 13 (3.1); HF, 5/57 (8.8)	Cut-off value, 100 ug/L； elevated patients, 30/174 (17.2%)	Cut-off value, 0.026 ng/mL； elevated patients, 29/345 (8.4%)	Death, discharge	9
Zhang G et al.[60]	2020.01.02 - 2020.02.10	Wuhan, China	221	55.0 (39.0-66.5)	108 (48.9)	55	12 (5.4)	CAD, 22 (10.0)	NA	Average level of cTnI,7.6 (3.6-21.5) pg/mL; cut-off value, 0.0262 ng/mL; elevated patients, 17 (7.7%)	Discharge, death, severe COVID-19	8
Zhang Q et al.[61]	2020.01.23-2020.02.25	Jilin, China	41	45 (31-53)	23 (56.1)	8 (19.5)	NA	CAD, 1 (2.4)	Average level of Mb, 26.0 (19.7-118.6) ug/L elevated patients, 11 (26.8%)	Average level of cTnI; 1.5 (0.8-5.0) ng/mL; elevated patients, 41 (100%)	Severe COVID-19	10
Zhang Y et al.[62]	2020.02.28 - 2020.04.04	Wuhan, China	166	62.7 (14.2) [b]	85 (51.2)	136 (81.9)	24 (14.5)	CAD, 30 (18.1); NT-proBNP, 179.0 (67.0-457.0) pg/mL	Average level of Mb,54.8 (33.8-127.2) ug/L; cut-off value, 106 ug/L； elevated patients, 28/166 (16.9%)	Average level of cTnI,5.0 (2.2-10.7) pg/mL; cut-off value, 0.0156 ng/mL; elevated patients, 17 /166 (10.2%)	Discharge, death	10
Zhao M et al.[63]	2020.01.01 - 2020.02.14	Wuhan, China	1000	61.0 (46.0-70.0)	466 (46.6)	615 (61.5)	119 (11.9)	CAD, 60 (6.0)	Average level of Mb, 44.54 (28.5-85.05) ug/L; cut-off value, 110 ug/L； elevated patients, 132/754 (17.5%)	Average level of cTnI,0.006 (0.006-0.018) ng/mL; cut-off value, 0.0796 ng/mL; elevated patients, 66/758 (8.7%)	Death, discharge	8
Zhao X et al.[64]	2020.01.16 - 2020.02.10	Hubei, China	91	46.0	49 (53.8)	30 (33.0)	2 (2.2)	HF, 14 (15.4)	NA	Cut-off value, 0.01 ng/mL; elevated patients, 3/88 (3.4%)	Death, discharge	9
Zhou F et al.[65]	2019.12.29 - 2020.01.31	Wuhan, China	191	56.0 (46.0-67.0)	119 (62.0)	66 (34.6)	54 (28.3)	CAD, 15 (8.0); HF, 44 (23.0)	NA	Average level of cTnI,4.1 (2.0-14.1) ng/mL; cut-off value, 28 ng/mL； elevated patients, 24/145 (16.6%)	Death, admission to ICU	10

No., number of COVID-19 confirmed patients; IQR, interquartile range; CSR, case severity rate; CFR, case fatality rate; Mb, myoglobin; cTnI, cardiac troponin I; NA, data not available.

[a] Study quality was assessed using the Quality Assessment Forms recommended by Agency for Healthcare Research and Quality (AHRQ);

[b] Mean (standard deviation);

[c] Median (range).

Figure S1. Forest plot of the overall prevalence of severe illness (A) and in-hospital mortality (B) and ICU-admission (C) of COVID-19 patients

A

Study	Events	Total	Proportion	95%-CI	Weight (fixed)	Weight (random)
Cai Q et al.	58	298	0.195	[0.151; 0.244]	2.7%	4.2%
Cao J et al.	153	244	0.627	[0.563; 0.688]	1.5%	4.2%
Cao M et al.	19	198	0.096	[0.059; 0.146]	3.3%	4.2%
Deng Q et al.	67	112	0.598	[0.501; 0.690]	0.7%	4.0%
Feng Y et al.	124	476	0.261	[0.222; 0.302]	3.5%	4.2%
Garibaldi BT et al.	171	832	0.206	[0.179; 0.235]	7.3%	4.2%
Han H et al.	75	273	0.275	[0.223; 0.332]	2.0%	4.2%
He F et al.	30	288	0.104	[0.071; 0.145]	4.4%	4.2%
He X et al.	501	1031	0.486	[0.455; 0.517]	5.9%	4.2%
Hu L et al.	172	323	0.533	[0.476; 0.588]	1.8%	4.2%
Huang J et al.	22	98	0.224	[0.146; 0.320]	0.8%	4.1%
Huang R et al.	23	202	0.114	[0.074; 0.166]	2.9%	4.2%
Li C et al.	476	2068	0.230	[0.212; 0.249]	16.6%	4.2%
Li X et al.	269	548	0.491	[0.448; 0.534]	3.1%	4.2%
Shen Y et al.	26	325	0.080	[0.053; 0.115]	6.3%	4.2%
Wei J et al.	37	101	0.366	[0.273; 0.468]	0.6%	4.0%
Wu Y et al.	2	125	0.016	[0.002; 0.057]	11.3%	4.2%
Xu P et al.	55	703	0.078	[0.059; 0.101]	13.9%	4.2%
Zhang G et al.	55	221	0.249	[0.193; 0.311]	1.7%	4.2%
Zhang Q et al.	8	41	0.195	[0.088; 0.349]	0.4%	3.9%
Zhang Y et al.	136	166	0.819	[0.752; 0.875]	1.6%	4.2%
Zhao M et al.	615	1000	0.615	[0.584; 0.645]	6.0%	4.2%
Zhao X et al.	30	91	0.330	[0.235; 0.436]	0.6%	4.0%
Zhou F et al.	66	191	0.346	[0.278; 0.418]	1.2%	4.1%
**Fixed effect model**		**9955**	**0.234**	**[0.227; 0.241]**	**100.0%**	**--**
**Random effects model**			**0.313**	**[0.232; 0.394]**	**--**	**100.0%**

Heterogeneity: $I^2 = 99\%$, $\tau^2 = 0.0404$, $p = 0$

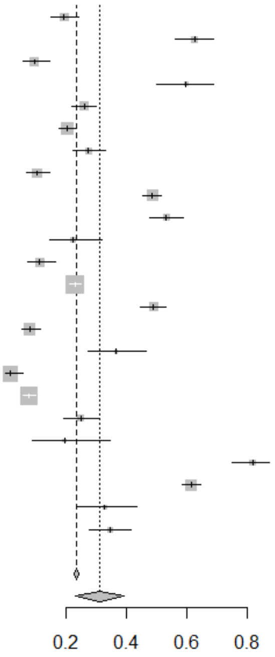

B

Study	Events	Total	Proportion	95%-CI	Weight (fixed)	Weight (random)
Arcari L et al.	23	111	0.207	[0.136; 0.295]	0.3%	1.7%
Bardají A et al.	37	186	0.199	[0.144; 0.264]	0.5%	1.8%
Bhatla A et al.	30	700	0.043	[0.029; 0.061]	0.3%	1.8%
Cai Q et al.	3	298	0.010	[0.002; 0.029]	0.0%	1.0%
Calvo-Fernández A et al.	104	872	0.119	[0.098; 0.143]	1.3%	1.9%
Cao J et al.	17	102	0.167	[0.100; 0.253]	0.2%	1.7%
Cao J et al.	14	244	0.057	[0.032; 0.094]	0.2%	1.6%
Chen N et al.	11	99	0.111	[0.057; 0.190]	0.1%	1.5%
Chorin E et al.	50	204	0.245	[0.188; 0.310]	0.7%	1.8%
Cipriani A et al.	20	109	0.183	[0.116; 0.269]	0.3%	1.7%
Deng Q et al.	14	112	0.125	[0.070; 0.201]	0.2%	1.6%
Elhadi M et al.	149	1207	0.123	[0.105; 0.143]	1.8%	1.9%
Feng Y et al.	38	476	0.080	[0.057; 0.108]	0.4%	1.8%
Ferguson J et al.	6	72	0.083	[0.031; 0.173]	0.1%	1.3%
Ferrante G et al.	68	332	0.205	[0.163; 0.252]	0.9%	1.9%
Franks C et al.	34	182	0.187	[0.133; 0.251]	0.4%	1.8%
García de Guadiana-Romualdo L et al.	187	1280	0.146	[0.127; 0.167]	2.3%	1.9%
Garibaldi BT et al.	131	832	0.157	[0.133; 0.184]	1.7%	1.9%
Guo T et al.	43	187	0.230	[0.172; 0.297]	0.6%	1.8%
Han H et al.	24	273	0.088	[0.057; 0.128]	0.3%	1.7%
Harmouch F et al.	81	560	0.145	[0.117; 0.177]	1.0%	1.9%
He F et al.	1	288	0.003	[0.000; 0.019]	0.0%	0.5%
He X et al.	165	1031	0.160	[0.138; 0.184]	2.1%	1.9%
Hu L et al.	35	323	0.108	[0.077; 0.147]	0.4%	1.8%
Huang C et al.	6	41	0.146	[0.056; 0.292]	0.1%	1.3%
Huang J et al.	1	98	0.010	[0.000; 0.056]	0.0%	0.5%
Lala A et al.	506	2736	0.185	[0.171; 0.200]	6.6%	1.9%
Li C et al.	183	2068	0.088	[0.077; 0.102]	2.1%	1.9%
Li X et al.	90	548	0.164	[0.134; 0.198]	1.1%	1.9%
Maeda T et al.	38	181	0.210	[0.153; 0.277]	0.5%	1.8%
Majure D et al.	1397	6247	0.224	[0.213; 0.234]	19.2%	1.9%
Manocha KK et al.	95	446	0.213	[0.176; 0.254]	1.3%	1.9%
Meng Y et al.	21	168	0.125	[0.079; 0.185]	0.3%	1.7%
Merugu GP et al.	23	217	0.106	[0.068; 0.155]	0.3%	1.7%
Mikami T et al.	806	3708	0.217	[0.204; 0.231]	11.0%	1.9%
Özyılmaz S et al.	9	105	0.086	[0.040; 0.156]	0.1%	1.5%
Palaiodimos L et al.	48	200	0.240	[0.183; 0.305]	0.7%	1.8%
Peiró ÓM et al.	37	196	0.189	[0.137; 0.251]	0.5%	1.8%
Price-Haywood E et al.	326	1382	0.236	[0.214; 0.259]	4.5%	1.9%
Qin J et al.	193	3219	0.060	[0.052; 0.069]	2.2%	1.9%
Richardson S et al.	553	2634	0.210	[0.195; 0.226]	7.5%	1.9%
Saleh S et al.	77	386	0.199	[0.161; 0.243]	1.0%	1.9%
Schiavone M et al.	105	674	0.156	[0.129; 0.185]	1.3%	1.9%
Shah P et al.	66	309	0.214	[0.169; 0.264]	0.9%	1.9%
Shen Y et al.	3	325	0.009	[0.002; 0.027]	0.0%	1.0%
Singh N et al.	26	276	0.094	[0.062; 0.135]	0.3%	1.7%
Stefanini G et al.	92	397	0.232	[0.191; 0.276]	1.3%	1.9%
Suleyman G et al.	74	463	0.160	[0.128; 0.196]	0.9%	1.9%
Tanboğa IH et al.	882	14855	0.059	[0.056; 0.063]	10.0%	1.9%
Tomasoni D et al.	163	692	0.236	[0.204; 0.269]	2.3%	1.9%
Wang D et al.	6	138	0.043	[0.016; 0.092]	0.1%	1.3%
Wang Z et al.	177	293	0.604	[0.546; 0.660]	4.8%	1.9%
Wei J et al.	3	101	0.030	[0.006; 0.084]	0.0%	1.0%
Xu P et al.	33	703	0.047	[0.033; 0.065]	0.4%	1.8%
Zeng J et al.	3	416	0.007	[0.001; 0.021]	0.0%	1.0%
Zhang G et al.	12	221	0.054	[0.028; 0.093]	0.1%	1.5%
Zhang Y et al.	24	166	0.145	[0.095; 0.207]	0.3%	1.7%
Zhao M et al.	119	1000	0.119	[0.100; 0.141]	1.4%	1.9%
Zhao X et al.	2	91	0.022	[0.003; 0.077]	0.0%	0.8%
Zhou F et al.	54	191	0.283	[0.220; 0.352]	0.8%	1.9%
**Fixed effect model**		**55971**	**0.172**	**[0.168; 0.175]**	**100.0%**	**--**
**Random effects model**			**0.125**	**[0.107; 0.146]**	**--**	**100.0%**

Heterogeneity: $I^2 = 98\%$, $\tau^2 = 0.3230$, $p = 0$

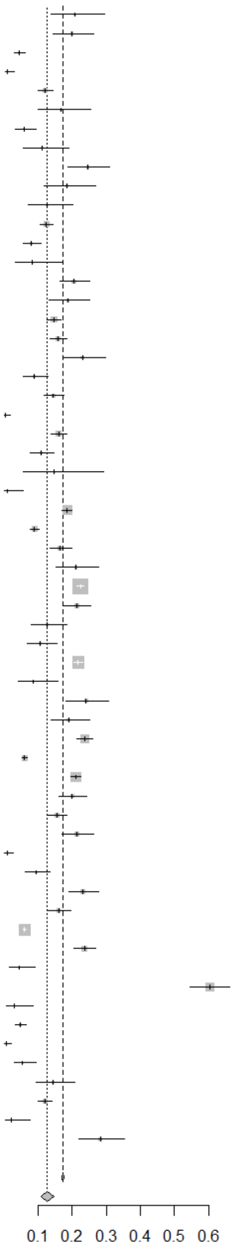

C

Study	Events	Total	Proportion	95%-CI	Weight (fixed)	Weight (random)
Bardaji´ A et al.	33	186	0.177	[0.125; 0.240]	0.6%	5.7%
Calvo-Fernández A et al.	58	872	0.067	[0.051; 0.085]	6.1%	6.1%
Cipriani A et al.	31	109	0.284	[0.202; 0.379]	0.2%	5.2%
García de Guadiana-Romualdo L et al.	249	1280	0.195	[0.173; 0.217]	3.6%	6.1%
Maeda T et al.	52	181	0.287	[0.223; 0.359]	0.4%	5.5%
Majure D et al.	1888	6247	0.302	[0.291; 0.314]	12.9%	6.1%
Manocha KK et al.	237	446	0.531	[0.484; 0.578]	0.8%	5.8%
Peiró ÓM et al.	34	196	0.173	[0.123; 0.234]	0.6%	5.7%
Saleh S et al.	76	386	0.197	[0.158; 0.240]	1.1%	5.9%
Schiavone M et al.	46	674	0.068	[0.050; 0.090]	4.6%	6.1%
Shah P et al.	110	309	0.356	[0.303; 0.412]	0.6%	5.7%
Singh N et al.	93	276	0.337	[0.281; 0.396]	0.5%	5.7%
Stefanini G et al.	65	397	0.164	[0.129; 0.204]	1.3%	6.0%
Tanboğa IH et al.	2020	14855	0.136	[0.131; 0.142]	55.2%	6.2%
Zeng J et al.	35	416	0.084	[0.059; 0.115]	2.4%	6.0%
Zhang Y et al.	7	166	0.042	[0.017; 0.085]	1.8%	6.0%
Zhao M et al.	63	1000	0.063	[0.049; 0.080]	7.4%	6.1%
**Fixed effect model**		**27996**	**0.152**	**[0.148; 0.156]**	**100.0%**	--
**Random effects model**			**0.201**	**[0.153; 0.249]**	--	**100.0%**

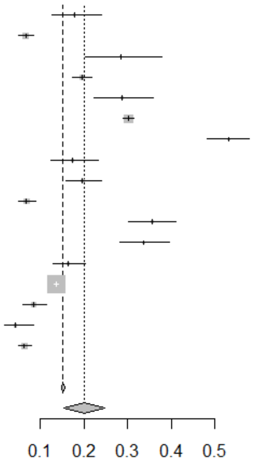

Heterogeneity: $I^2 = 99\%$, $\tau^2 = 0.0097$, $p < 0.01$

Figure S2. Forest plot showing result of sensitivity analysis after excluding each study in turn

A. Sensitivity analysis of the prevalence of elevated Mb in the general population

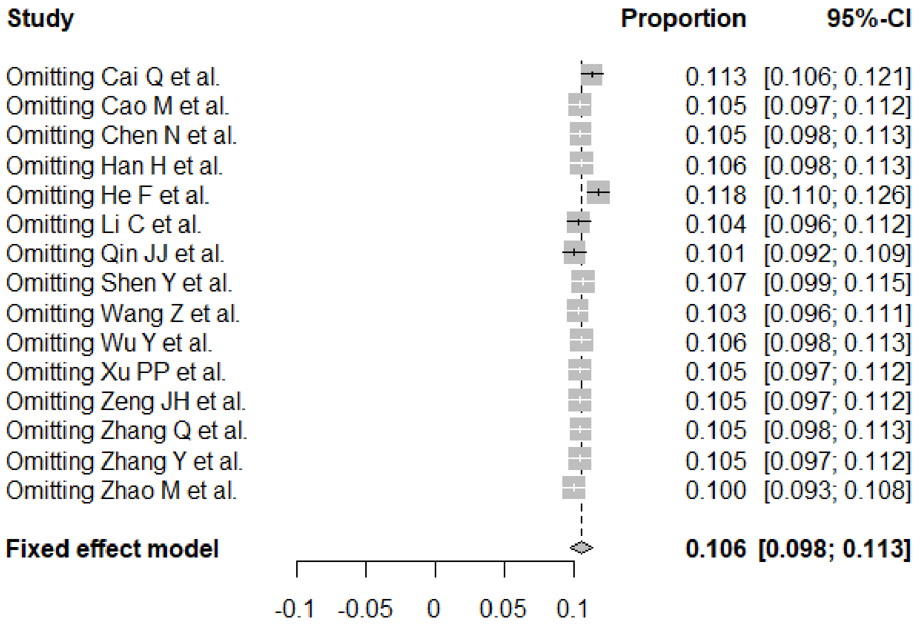

B. Sensitivity analysis of the prevalence of elevated cTnI in the general population

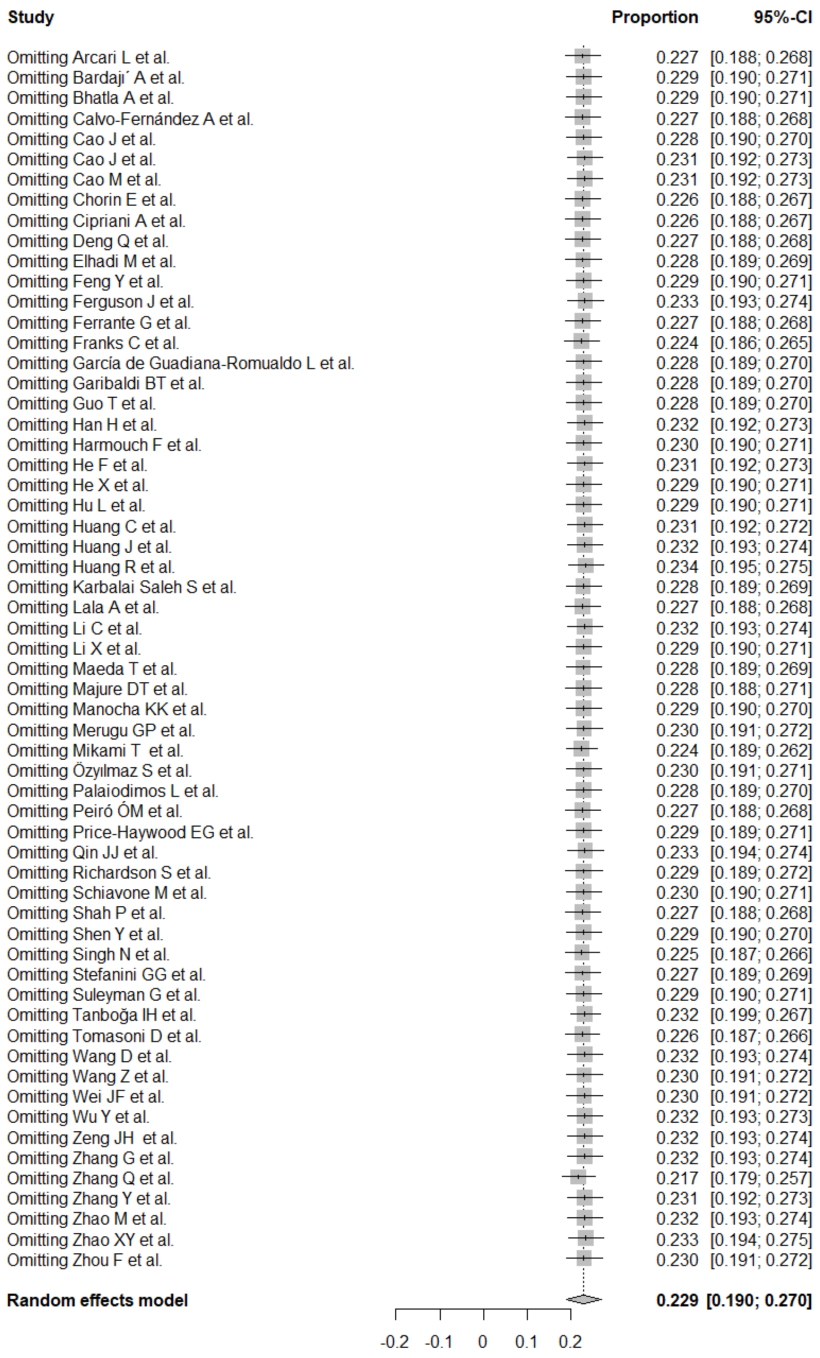

C. Sensitivity analysis of the incidence of severe illness

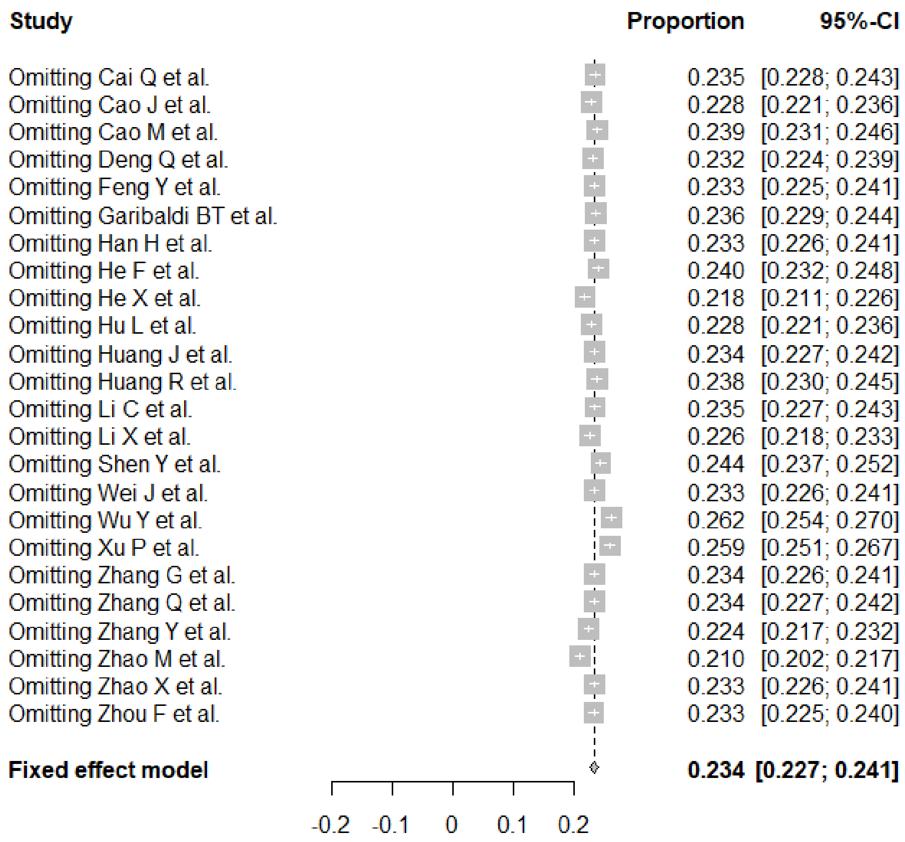

D. Sensitivity analysis of the incidence of in-hospital mortality

Study	Proportion	95%-CI
Omitting Arcari L et al.	0.124	[0.106; 0.145]
Omitting Bardají A et al.	0.124	[0.106; 0.145]
Omitting Bhatla A et al.	0.127	[0.109; 0.149]
Omitting Cai Q et al.	0.128	[0.110; 0.149]
Omitting Calvo-Fernández A et al.	0.125	[0.107; 0.146]
Omitting Cao J et al.	0.124	[0.106; 0.145]
Omitting Cao J et al.	0.126	[0.108; 0.148]
Omitting Chen N et al.	0.125	[0.107; 0.146]
Omitting Chorin E et al.	0.123	[0.105; 0.144]
Omitting Cipriani A et al.	0.124	[0.106; 0.145]
Omitting Deng Q et al.	0.125	[0.107; 0.146]
Omitting Elhadi M et al.	0.125	[0.107; 0.146]
Omitting Feng Y et al.	0.126	[0.108; 0.147]
Omitting Ferguson J et al.	0.125	[0.107; 0.147]
Omitting Ferrante G et al.	0.124	[0.106; 0.145]
Omitting Franks C et al.	0.124	[0.106; 0.145]
Omitting García de Guadiana-Romualdo L et al.	0.124	[0.106; 0.145]
Omitting Garibaldi BT et al.	0.124	[0.106; 0.145]
Omitting Guo T et al.	0.123	[0.105; 0.144]
Omitting Han H et al.	0.126	[0.107; 0.147]
Omitting Harmouch F et al.	0.124	[0.106; 0.145]
Omitting He F et al.	0.127	[0.109; 0.148]
Omitting He X et al.	0.124	[0.106; 0.145]
Omitting Hu L et al.	0.125	[0.107; 0.146]
Omitting Huang C et al.	0.125	[0.107; 0.146]
Omitting Huang J et al.	0.126	[0.108; 0.148]
Omitting Lala A et al.	0.123	[0.105; 0.145]
Omitting Li C et al.	0.126	[0.108; 0.147]
Omitting Li X et al.	0.124	[0.106; 0.145]
Omitting Maeda T et al.	0.124	[0.106; 0.145]
Omitting Majure D et al.	0.122	[0.103; 0.144]
Omitting Manocha KK et al.	0.123	[0.105; 0.144]
Omitting Meng Y et al.	0.125	[0.107; 0.146]
Omitting Merugu GP et al.	0.125	[0.107; 0.146]
Omitting Mikami T et al.	0.122	[0.104; 0.144]
Omitting Özyılmaz S et al.	0.126	[0.107; 0.147]
Omitting Palaiodimos L et al.	0.123	[0.105; 0.144]
Omitting Peiró ÓM et al.	0.124	[0.106; 0.145]
Omitting Price-Haywood E et al.	0.123	[0.105; 0.144]
Omitting Qin J et al.	0.127	[0.109; 0.148]
Omitting Richardson S et al.	0.123	[0.104; 0.144]
Omitting Saleh S et al.	0.124	[0.106; 0.145]
Omitting Schiavone M et al.	0.124	[0.106; 0.145]
Omitting Shah P et al.	0.123	[0.106; 0.144]
Omitting Shen Y et al.	0.128	[0.110; 0.150]
Omitting Singh N et al.	0.125	[0.107; 0.147]
Omitting Stefanini G et al.	0.123	[0.105; 0.144]
Omitting Suleyman G et al.	0.124	[0.106; 0.145]
Omitting Tanboğa IH et al.	0.132	[0.116; 0.150]
Omitting Tomasoni D et al.	0.123	[0.105; 0.144]
Omitting Wang D et al.	0.127	[0.108; 0.148]
Omitting Wang Z et al.	0.124	[0.107; 0.142]
Omitting Wei J et al.	0.127	[0.108; 0.148]
Omitting Xu P et al.	0.127	[0.109; 0.149]
Omitting Zeng J et al.	0.128	[0.110; 0.150]
Omitting Zhang G et al.	0.127	[0.108; 0.148]
Omitting Zhang Y et al.	0.124	[0.106; 0.146]
Omitting Zhao M et al.	0.125	[0.107; 0.146]
Omitting Zhao X et al.	0.127	[0.108; 0.148]
Omitting Zhou F et al.	0.123	[0.105; 0.144]
**Random effects model**	**0.125**	**[0.107; 0.146]**

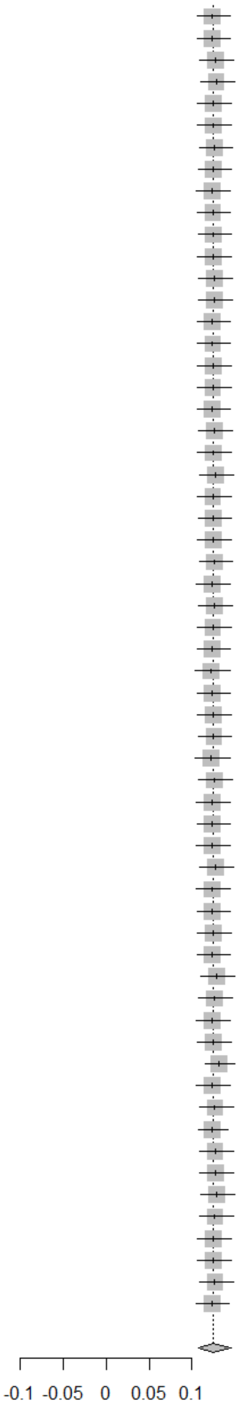

E. Sensitivity analysis of the incidence of ICU-admission

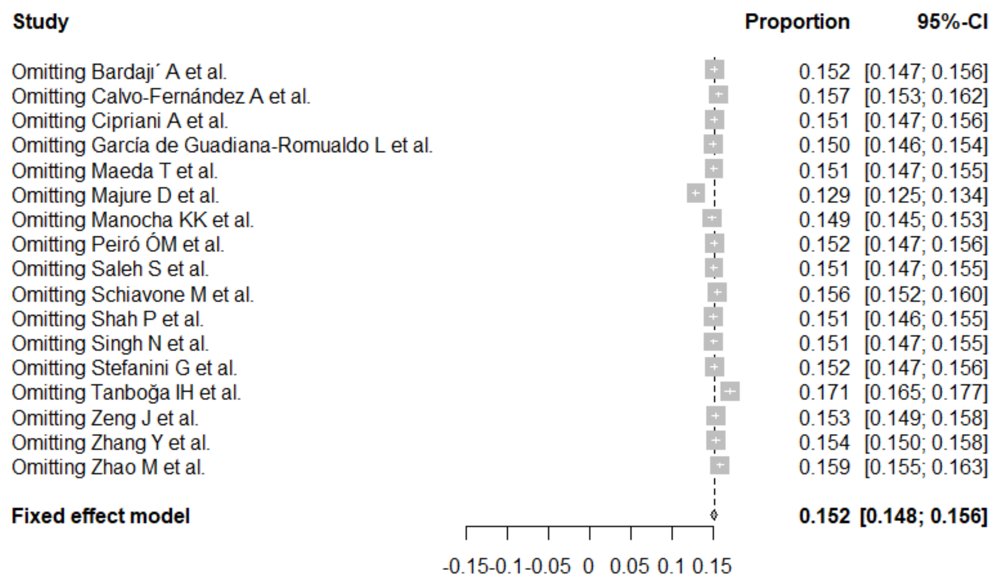

F. Sensitivity analysis of the prevalence of elevated Mb in severe disease group

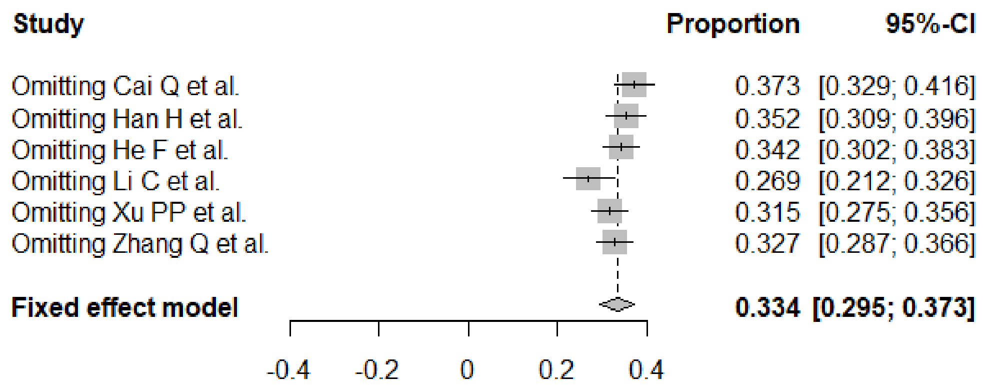

G. Sensitivity analysis of the prevalence of elevated cTnI in severe disease group

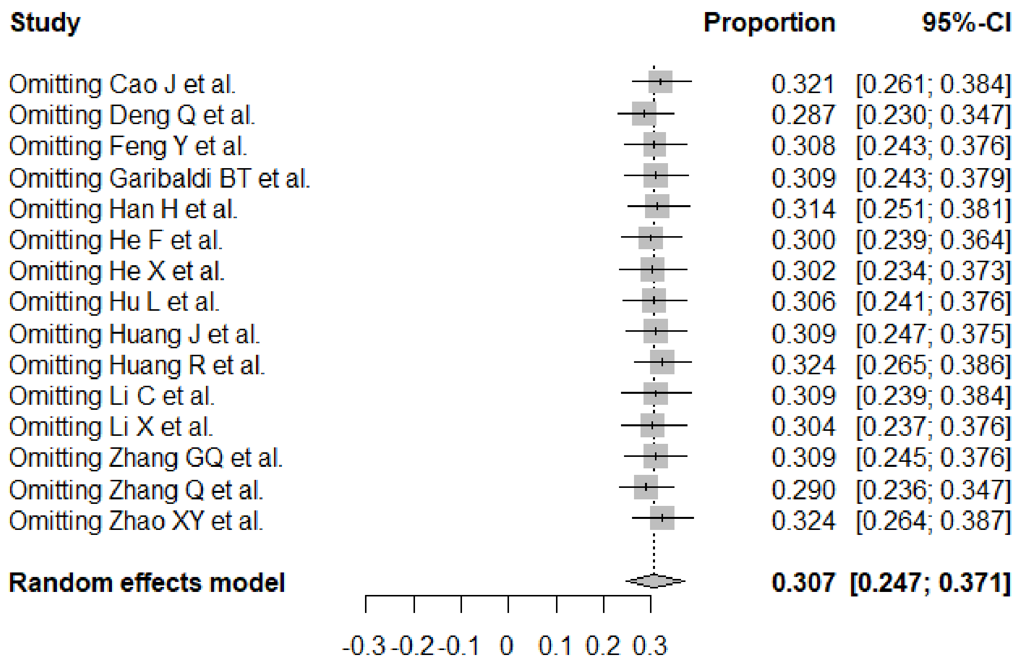

H. Sensitivity analysis of the prevalence of elevated Mb in non-survivor group

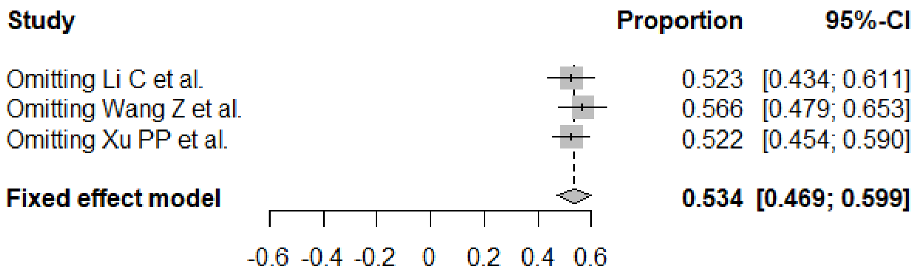

I. Sensitivity analysis of the prevalence of elevated cTnI in non-survivor group

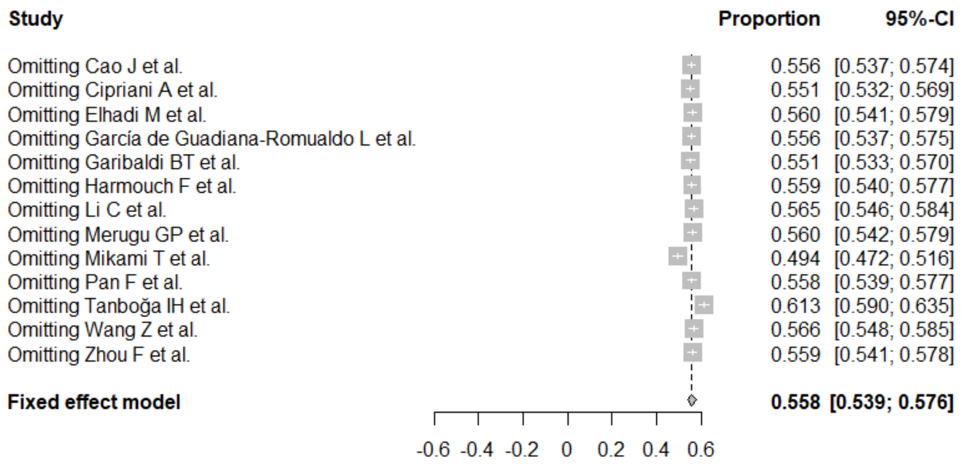

J. Sensitivity analysis of the risk of elevated Mb for severe illness

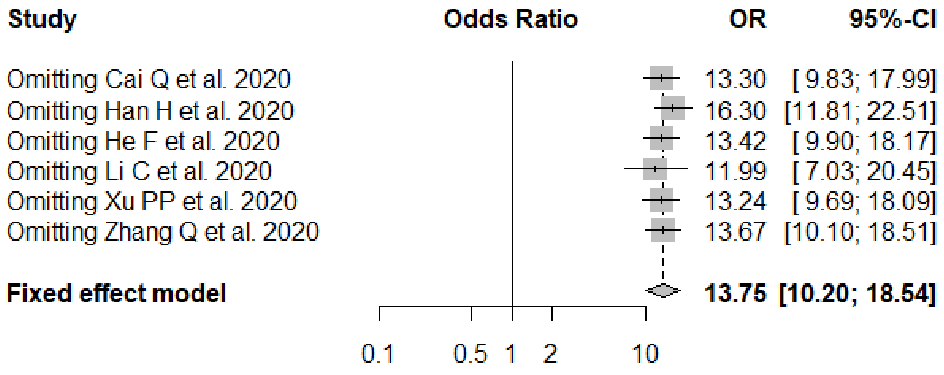

K. Sensitivity analysis of the risk of elevated cTnI for severe illness

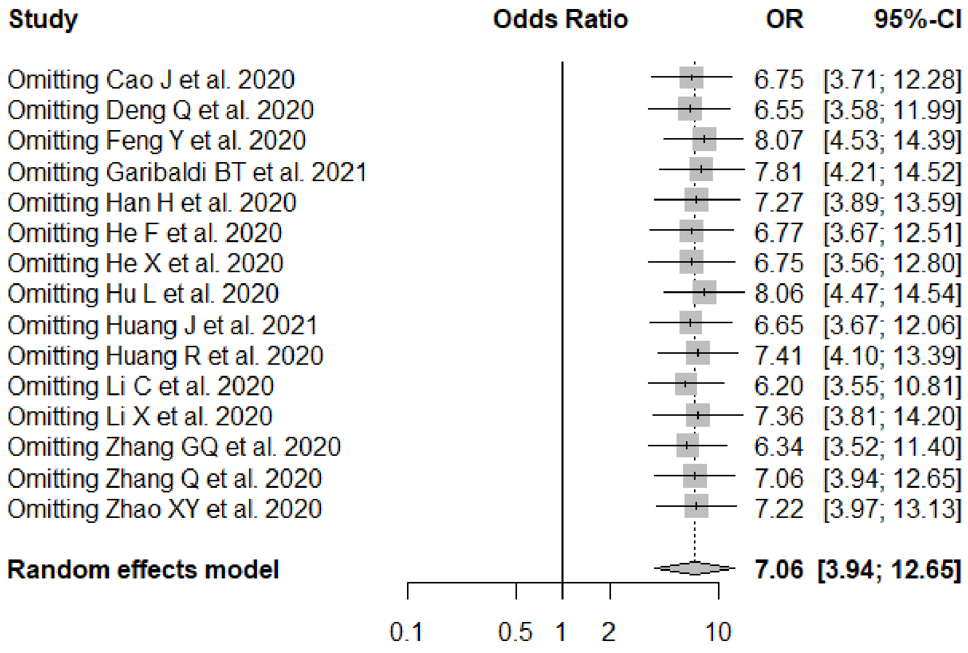

L. Sensitivity analysis of the risk of elevated Mb for in-hospital mortality

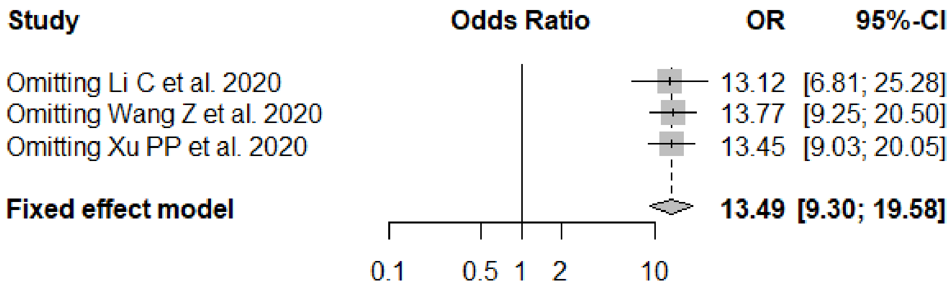

M. Sensitivity analysis of the risk of elevated cTnI for in-hospital mortality

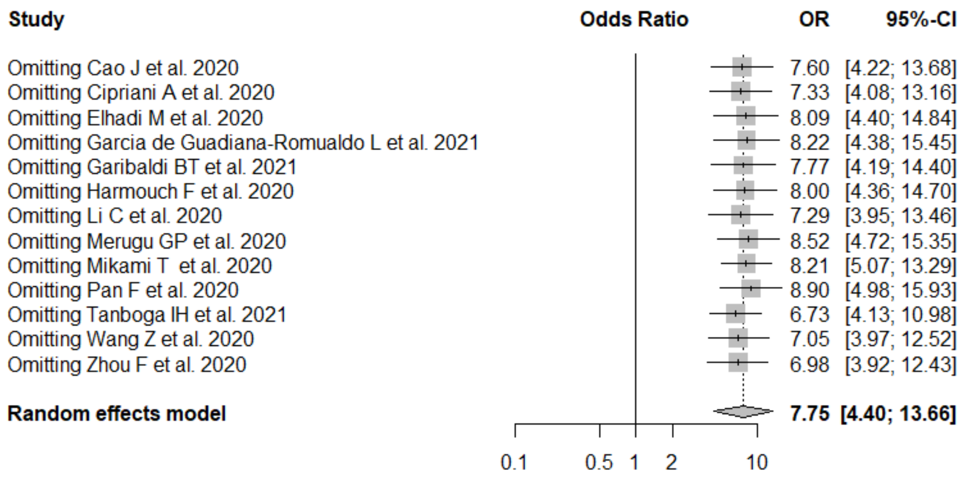

Figure S3. Funnel plot

A. Funnel plot of the prevalence of elevated Mb in the general population

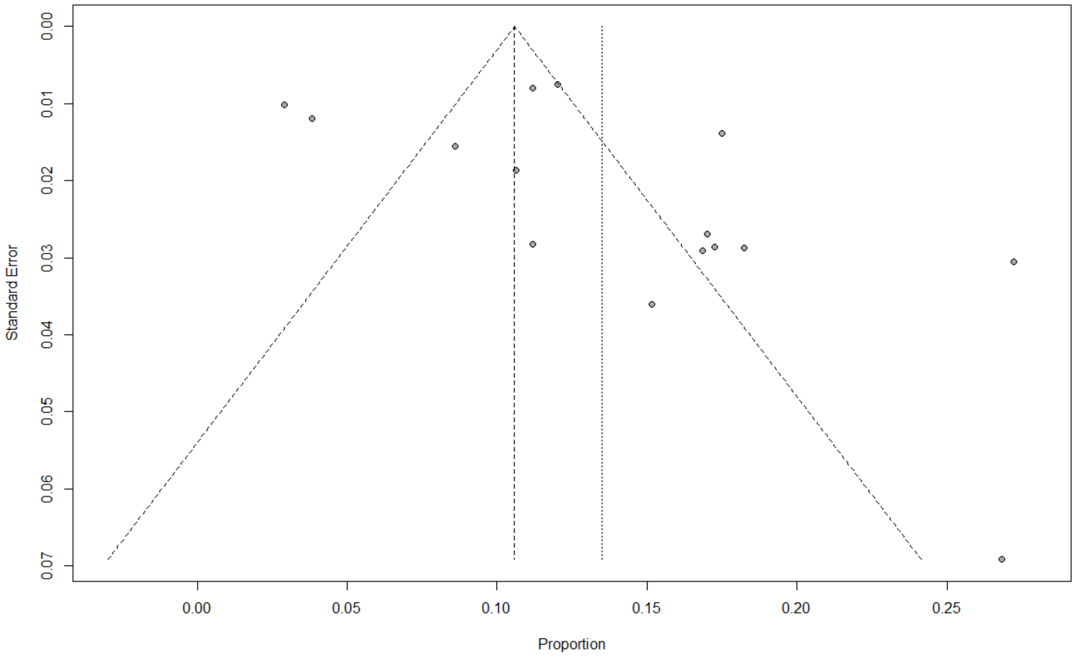

Egger p-value: 0.09453; Begger p-value: 0.08326; Peter p-value: 0.08192

B. Funnel plot of the prevalence of elevated cTnI in the general population

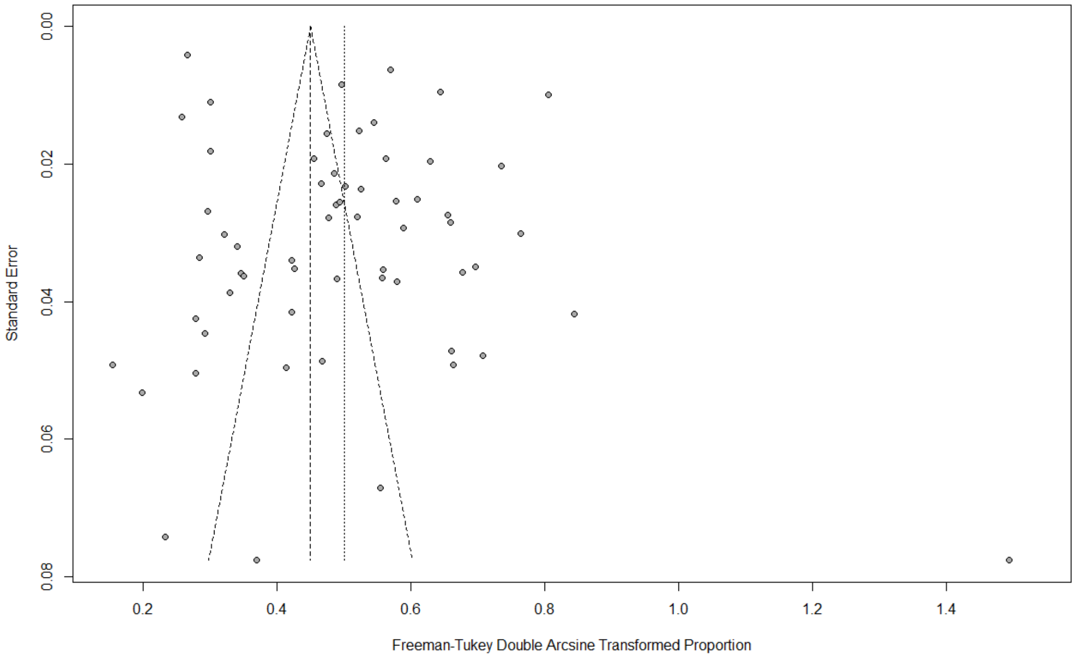

Egger p-value: 0.03789; Begger p-value: 0.07207; Peter p-value: 0.5637

C. Funnel plot of the incidence of severe illness

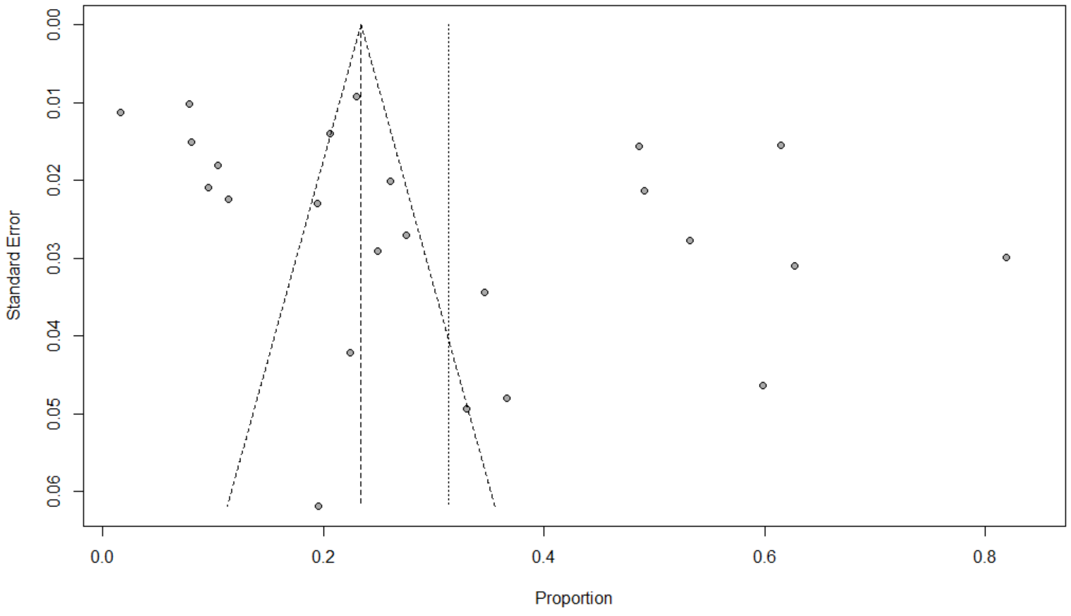

Egger p-value: 0.02466; Begger p-value: 0.01726; Peter p-value: 0.7713

D. Funnel plot of the incidence of in-hospital mortality

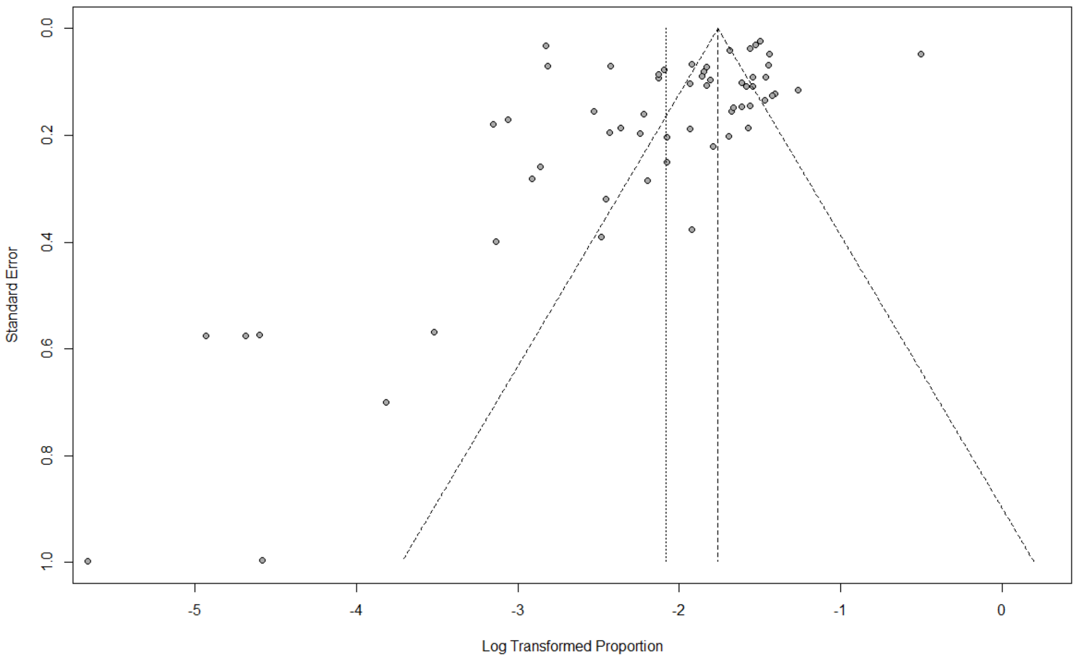

Egger p-value: 0.04792; Begger p-value: 0.0003063; Peter p-value: 0.4989

E. Funnel plot of the incidence of ICU-admission

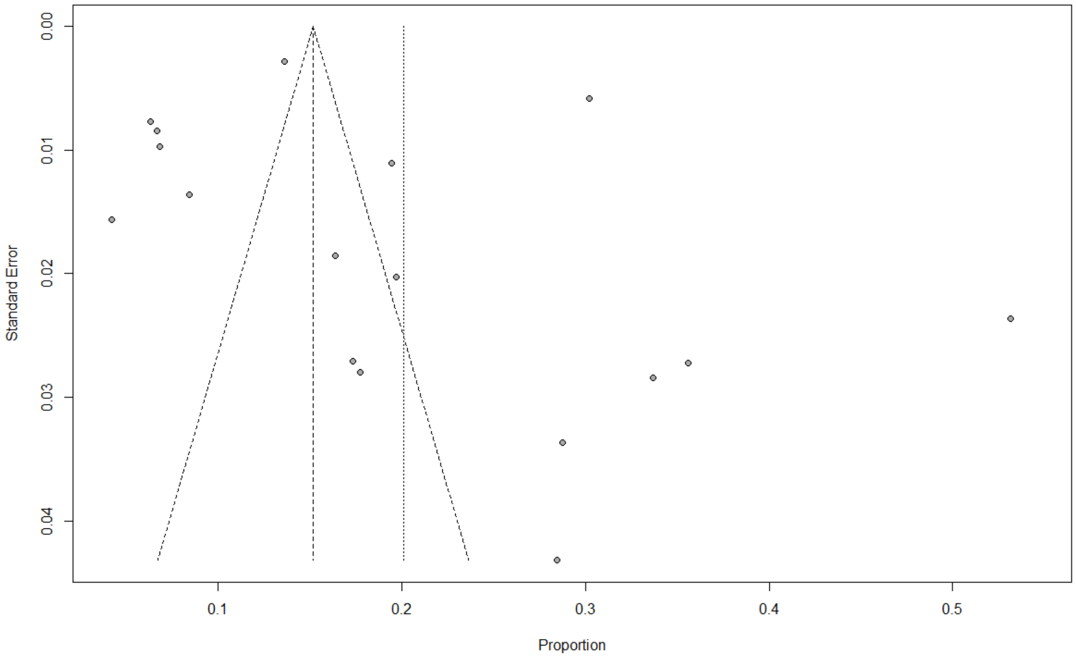

Egger p-value: 0.3967; Begger p-value: 0.02612; Peter p-value: 0.4039

F. Funnel plot of the prevalence of elevated Mb in severe disease group

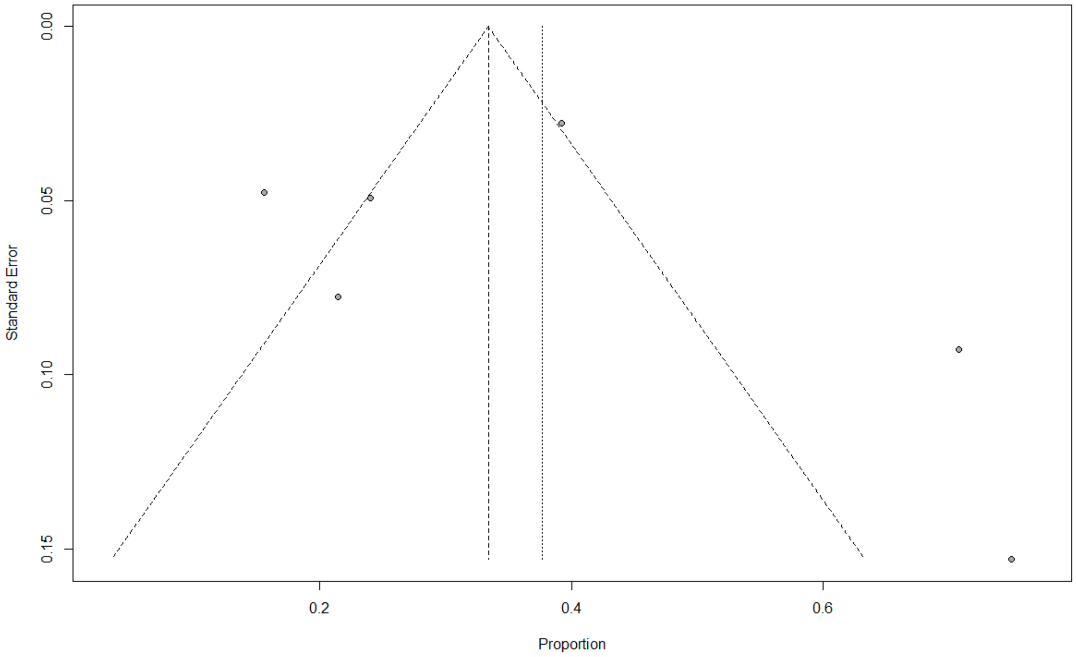

Egger p-value: 0.6935; Begger p-value: 0.3746; Peter p-value: 0.5328

G. Funnel plot of the prevalence of elevated cTnI in severe disease group

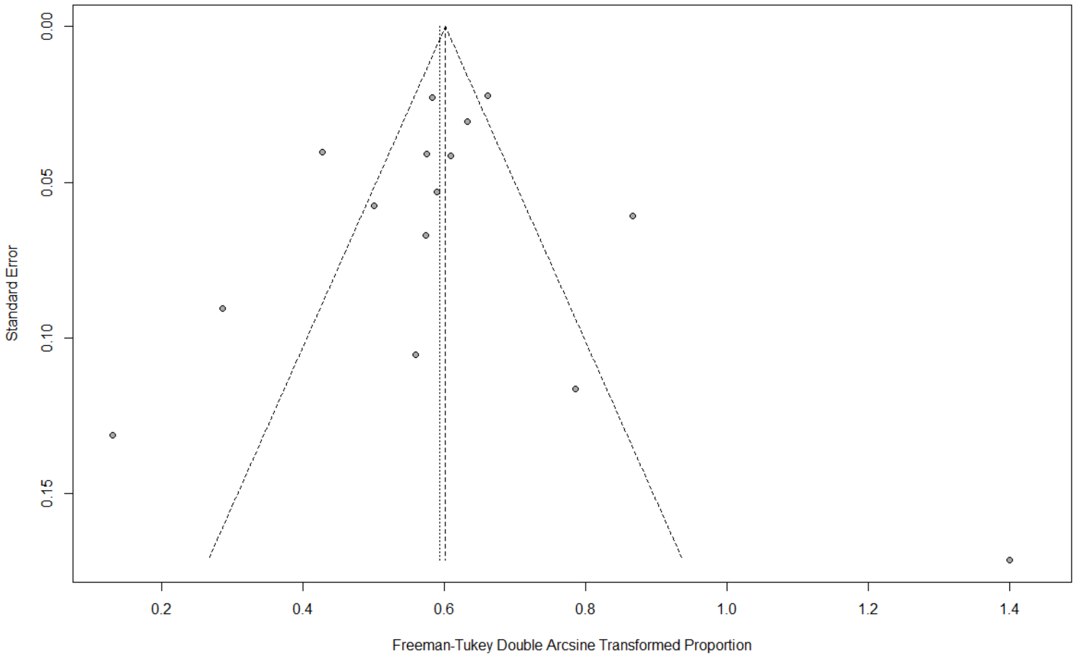

Egger p-value: 0.8258; Begger p-value: 0.882; Peter p-value: 0.8568

H. Funnel plot of the prevalence of elevated Mb in non-survivor group

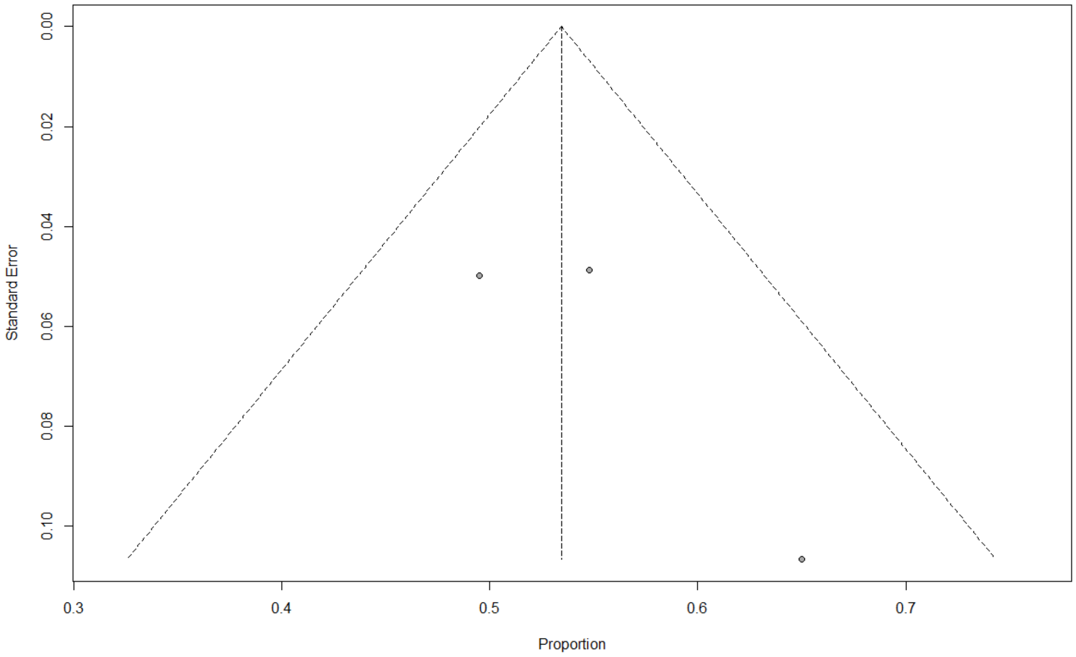

Egger p-value: 0.3916; Begger p-value: 0.6015; Peter p-value: 0.4063

I. Funnel plot of the prevalence of elevated cTnI in non-survivor group

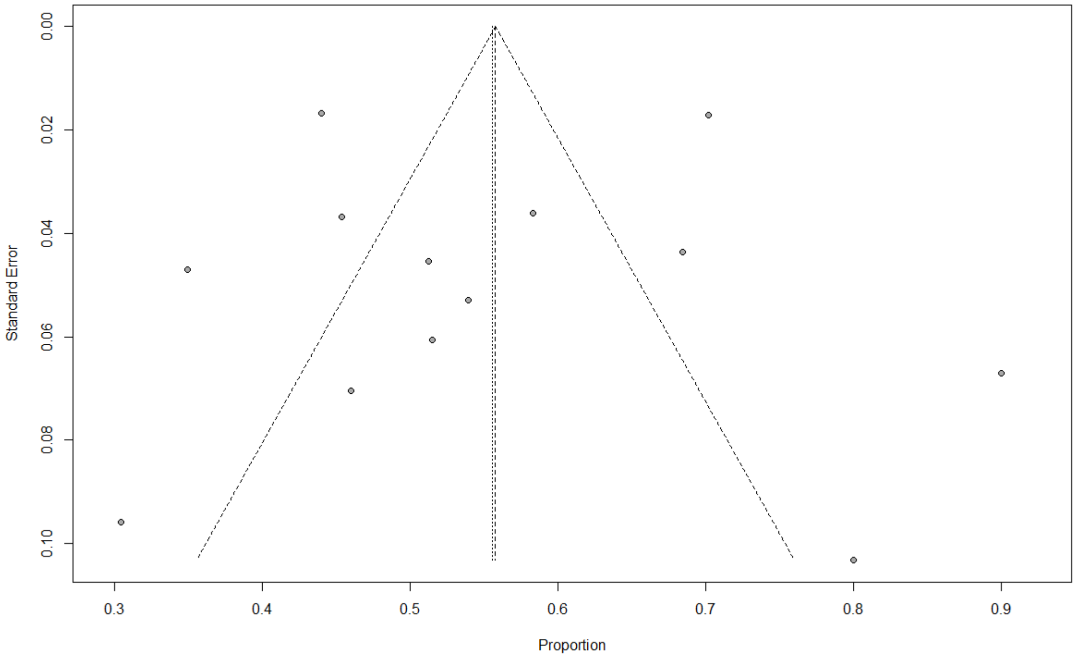

Egger p-value: 0.9162; Begger p-value: 0.9029; Peter p-value: 0.9438

J. Funnel plot of the risk of elevated Mb for severe illness

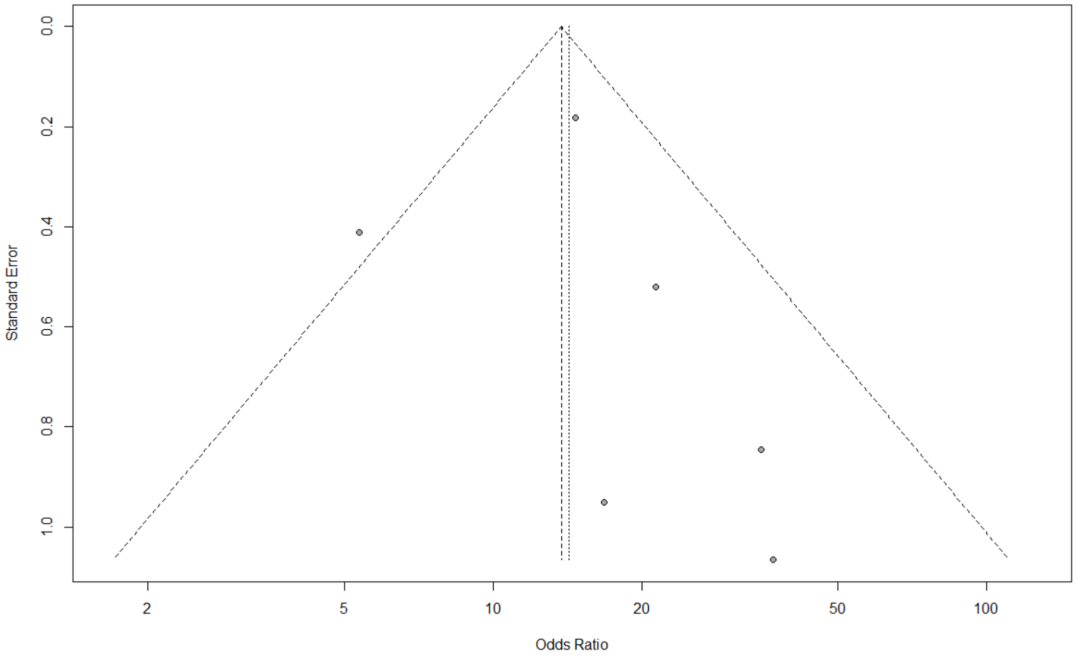

Egger p-value: 0.6346; Begger p-value: 0.3476; Peter p-value: 0.8624

K. Funnel plot of the risk of elevated cTnI for severe illness

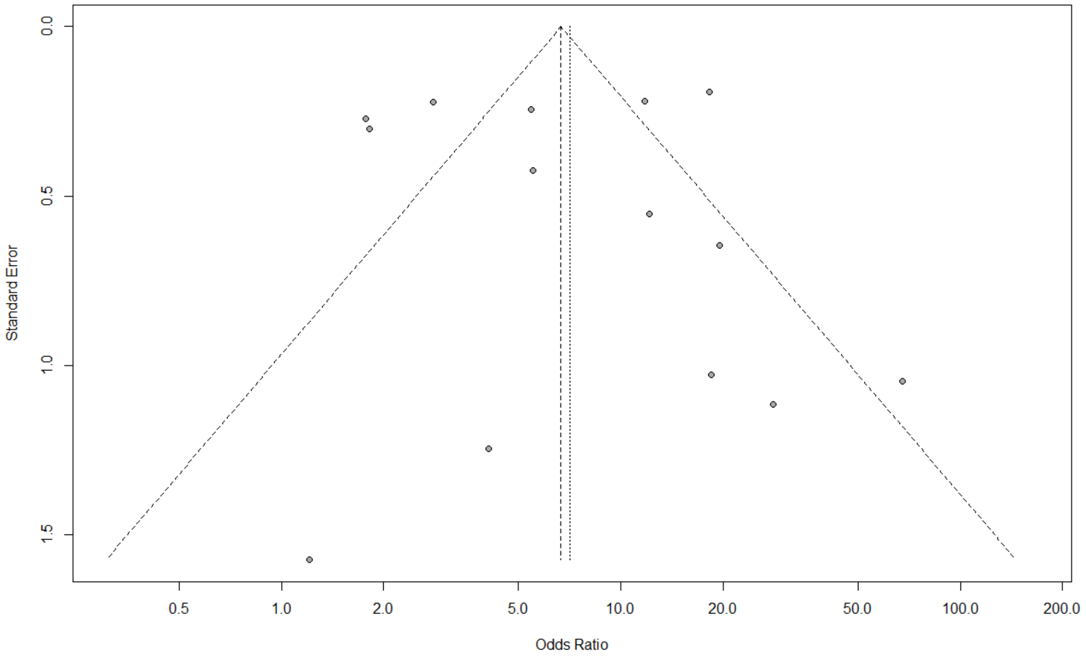

Egger p-value: 0.8127; Begger p-value: 0.8695; Peter p-value: 0.7997

L. Funnel plot of the risk of elevated Mb for in-hospital mortality

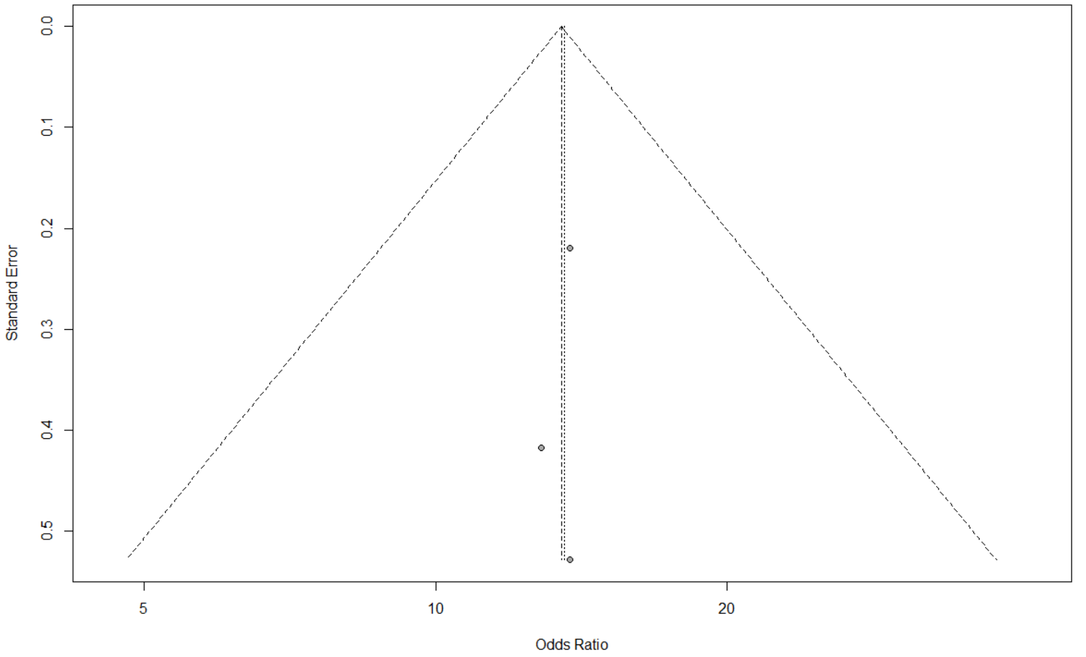

Egger p-value: 0.6612; Begger p-value: 0.6015; Peter p-value: 0.567

M. Funnel plot of the risk of elevated cTnI for in-hospital mortality

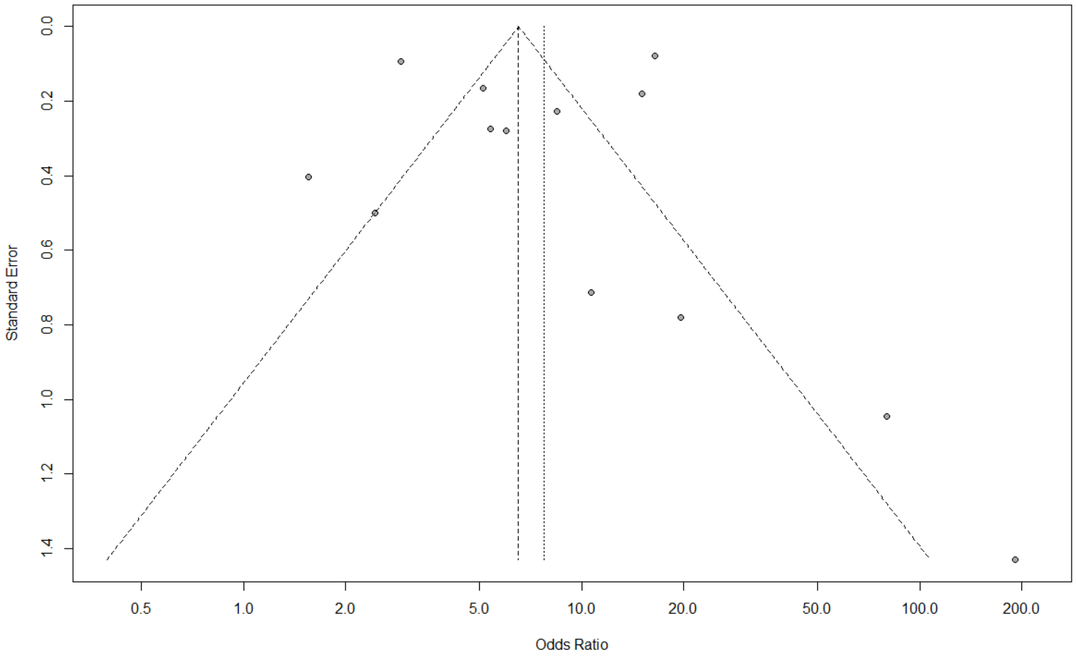

Egger p-value: 0.8723; Begger p-value: 0.2721; Peter p-value: 0.9568

References

1. Huang C, Wang Y, Li X, et al. Clinical features of patients infected with 2019 novel coronavirus in Wuhan, China. *Lancet (London, England)* 2020; **395**(10223): 497-506.

2. Metlay J, Waterer G, Long A, et al. Diagnosis and Treatment of Adults with Community-acquired Pneumonia. An Official Clinical Practice Guideline of the American Thoracic Society and Infectious Diseases Society of America. *American journal of respiratory and critical care medicine* 2019; **200**(7): e45-e67.

3. Arcari L, Luciani M, Cacciotti L, et al. Incidence and determinants of high-sensitivity troponin and natriuretic peptides elevation at admission in hospitalized COVID-19 pneumonia patients. *Intern Emerg Med* 2020.

4. Bardají A, Carrasquer A, Sánchez-Giménez R, et al. Prognostic implications of myocardial injury in patients with and without COVID-19 infection treated in a university hospital. 2021; **74**(1): 24-32.

5. Bhatla A, Mayer MM, Adusumalli S, et al. COVID-19 and cardiac arrhythmias. *Heart Rhythm* 2020.

6. Cai Q, Huang D, Ou P, et al. COVID-19 in a designated infectious diseases hospital outside Hubei Province, China. *Allergy* 2020; **75**(7): 1742-52.

7. Calvo-Fernández A, Izquierdo A, Subirana I, et al. Markers of myocardial injury in the prediction of short-term COVID-19 prognosis. 2021; **74**(7): 576-83.

8. Cao J, Tu WJ, Cheng W, et al. Clinical Features and Short-term Outcomes of 102 Patients with Corona Virus Disease 2019 in Wuhan, China. *Clin Infect Dis* 2020.

9. Cao J, Zheng Y, Luo Z, et al. Myocardial injury and COVID-19: Serum hs-cTnI level in risk stratification and the prediction of 30-day fatality in COVID-19 patients with no prior cardiovascular disease. *Theranostics* 2020; **10**(21): 9663-73.

10. Cao M, Zhang D, Wang Y, et al. Clinical Features of Patients Infected with the 2019 Novel Coronavirus (COVID-19) in Shanghai, China. *medRxiv : the preprint server for health sciences* 2020.

11. Chen N, Zhou M, Dong X, et al. Epidemiological and clinical characteristics of 99 cases of 2019 novel coronavirus pneumonia in Wuhan, China: a descriptive study. *The Lancet* 2020; **395**(10223): 507-13.

12. Chorin E, Dai M, Kogan E, et al. Electrocardiographic Risk Stratification in COVID-19 Patients. 2021; **8**: 636073.

13. Cipriani A, Capone F, Donato F, et al. Cardiac injury and mortality in patients with Coronavirus disease 2019 (COVID-19): insights from a mediation analysis. *Intern Emerg Med* 2020.

14. Deng Q, Hu B, Zhang Y, et al. Suspected myocardial injury in patients with COVID-19: Evidence from front-line clinical observation in Wuhan, China. *Int J Cardiol* 2020; **311**: 116-21.

15. Elhadi M, Momen A, Alsoufi A, et al. Epidemiological and clinical presentations of hospitalized COVID-19 patients in Libya: An initial report from Africa. 2021; **42**: 102064.

16. Feng Y. COVID-19 with Different Severity: A Multi-center Study of Clinical Features. 2020.

17. Ferguson J, Rosser JI, Quintero O, et al. Characteristics and Outcomes of Coronavirus Disease Patients under Nonsurge Conditions, Northern California, USA, March-April 2020. *Emerging Infect Dis* 2020; **26**(8).

18. Ferrante G, Fazzari F, Cozzi O, et al. Risk factors for myocardial injury and death in patients with COVID-19: insights from a cohort study with chest computed tomography. *Cardiovasc Res*

2020.
19. Franks CE, Scott MG, Farnsworth CW. Elevated Cardiac Troponin I Is Associated with Poor Outcomes in COVID-19 Patients at an Academic Medical Center in Midwestern USA. *J Appl Lab Med* 2020.
20. García de Guadiana-Romualdo L, Morell-García D, Rodríguez-Fraga O, et al. Cardiac troponin and COVID-19 severity: Results from BIOCOVID study. 2021; **51**(6): e13532.
21. Garibaldi B, Fiksel J, Muschelli J, et al. Patient Trajectories Among Persons Hospitalized for COVID-19 : A Cohort Study. 2021; **174**(1): 33-41.
22. Guo T, Fan Y, Chen M, et al. Cardiovascular Implications of Fatal Outcomes of Patients With Coronavirus Disease 2019 (COVID-19). *JAMA Cardiol* 2020.
23. Han H, Xie L, Liu R, et al. Analysis of heart injury laboratory parameters in 273 COVID-19 patients in one hospital in Wuhan, China. *J Med Virol* 2020; **92**(7): 819-23.
24. Harmouch F, Shah K, Hippen JT, Kumar A, Goel H. Is it all in the heart? Myocardial injury as major predictor of mortality among hospitalized COVID-19 patients. *J Med Virol* 2020.
25. Feng Y, Ling Y, Bai T, et al. COVID-19 with Different Severities: A Multicenter Study of Clinical Features. *American journal of respiratory and critical care medicine* 2020; **201**(11): 1380-8.
26. He X, Wang L, Wang H, et al. Factors associated with acute cardiac injury and their effects on mortality in patients with COVID-19. 2020; **10**(1): 20452.
27. Hu L, Chen S, Fu Y, et al. Risk Factors Associated with Clinical Outcomes in 323 COVID-19 Hospitalized Patients in Wuhan, China. *Clin Infect Dis* 2020.
28. Huang C, Wang Y, Li X, et al. Clinical features of patients infected with 2019 novel coronavirus in Wuhan, China. *The Lancet* 2020; **395**(10223): 497-506.
29. Huang J, Gao J, Zhu W, et al. Indicators and prediction models for the severity of Covid-19. 2021: e14571.
30. Huang R, Zhu L, Xue L, et al. Clinical findings of patients with coronavirus disease 2019 in Jiangsu province, China: A retrospective, multi-center study. *PLoS Negl Trop Dis* 2020; **14**(5): e0008280.
31. Karbalai Saleh S, Oraii A, Soleimani A, et al. The association between cardiac injury and outcomes in hospitalized patients with COVID-19. *Intern Emerg Med* 2020.
32. Lala A, Johnson KW, Januzzi JL, et al. Prevalence and Impact of Myocardial Injury in Patients Hospitalized With COVID-19 Infection. *J Am Coll Cardiol* 2020; **76**(5): 533-46.
33. Li C, Jiang J, Wang F, et al. Longitudinal correlation of biomarkers of cardiac injury, inflammation, and coagulation to outcome in hospitalized COVID-19 patients. *J Mol Cell Cardiol* 2020; **147**: 74-87.
34. Li X, Xu S, Yu M, et al. Risk factors for severity and mortality in adult COVID-19 inpatients in Wuhan. *J Allergy Clin Immunol* 2020; **146**(1): 110-8.
35. Maeda T, Obata R, Rizk D, Kuno TJH, lung, circulation. Cardiac Injury and Outcomes of Patients With COVID-19 in New York City. 2021; **30**(6): 848-53.
36. Majure DT, Gruberg L, Saba SG, et al. Usefulness of Elevated Troponin to Predict Death in Patients With COVID-19 and Myocardial Injury. *Am J Cardiol* 2020.
37. Manocha K, Kirzner J, Ying X, et al. Troponin and Other Biomarker Levels and Outcomes Among Patients Hospitalized With COVID-19: Derivation and Validation of the HAT COVID-19 Mortality Risk Score. 2021; **10**(6): e018477.
38. Merugu G, Nesheiwat Z, Balla M, et al. Predictors of mortality in 217 COVID-19 patients in

Northwest Ohio, United States: A retrospective study. 2021; **93**(5): 2875-82.
39. Mikami T, Miyashita H, Yamada T, et al. Risk Factors for Mortality in Patients with COVID-19 in New York City. *J Gen Intern Med* 2020.
40. Özyılmaz S, Ergün Alış E, Ermiş E, Allahverdiyev S, Uçar HJM. Assessment of the Relationship between Mortality and Troponin I Levels in Hospitalized Patients with the Novel Coronavirus (COVID-19). 2020; **56**(12).
41. Palaiodimos L, Kokkinidis DG, Li W, et al. Severe obesity, increasing age and male sex are independently associated with worse in-hospital outcomes, and higher in-hospital mortality, in a cohort of patients with COVID-19 in the Bronx, New York. *Metabolism* 2020; **108**: 154262.
42. Peiró Ó, Carrasquer A, Sánchez-Gimenez R, et al. Biomarkers and short-term prognosis in COVID-19. 2021; **26**(2): 119-26.
43. Price-Haywood EG, Burton J, Fort D, Seoane L. Hospitalization and Mortality among Black Patients and White Patients with Covid-19. *N Engl J Med* 2020; **382**(26): 2534-43.
44. Qin JJ, Cheng X, Zhou F, et al. Redefining Cardiac Biomarkers in Predicting Mortality of Inpatients With COVID-19. *Hypertension* 2020; **76**(4): 1104-12.
45. Richardson S, Hirsch JS, Narasimhan M, et al. Presenting Characteristics, Comorbidities, and Outcomes Among 5700 Patients Hospitalized With COVID-19 in the New York City Area. *JAMA* 2020.
46. Schiavone M, Gasperetti A, Mancone M, et al. Redefining the Prognostic Value of High-Sensitivity Troponin in COVID-19 Patients: The Importance of Concomitant Coronary Artery Disease. *J Clin Med* 2020; **9**(10).
47. Shah P, Doshi R, Chenna A, et al. Prognostic Value of Elevated Cardiac Troponin I in Hospitalized Covid-19 Patients. 2020; **135**: 150-3.
48. Shen Y, Zheng F, Sun D, et al. Epidemiology and clinical course of COVID-19 in Shanghai, China. *Emerging Microbes & Infections* 2020; **9**(1): 1537-45.
49. Singh N, Anchan RK, Besser SA, et al. High Sensitivity Troponin-T for Prediction of Adverse Events in Patients with COVID-19. *Biomarkers* 2020: 1-26.
50. Stefanini GG, Chiarito M, Ferrante G, et al. Early detection of elevated cardiac biomarkers to optimise risk stratification in patients with COVID-19. *Heart* 2020; **106**(19): 1512-8.
51. Suleyman G, Fadel RA, Malette KM, et al. Clinical Characteristics and Morbidity Associated With Coronavirus Disease 2019 in a Series of Patients in Metropolitan Detroit. *JAMA Netw Open* 2020; **3**(6): e2012270.
52. Tanboğa I, Canpolat U, Özcan Çetin E, et al. The prognostic role of cardiac troponin in hospitalized COVID-19 patients. 2021; **325**: 83-8.
53. Tomasoni D, Inciardi R, Lombardi C, et al. Impact of heart failure on the clinical course and outcomes of patients hospitalized for COVID-19. Results of the Cardio-COVID-Italy multicentre study. 2020; **22**(12): 2238-47.
54. Wang D, Hu B, Hu C, et al. Clinical Characteristics of 138 Hospitalized Patients With 2019 Novel Coronavirus-Infected Pneumonia in Wuhan, China. *JAMA* 2020.
55. Wang Z, Ye D, Wang M, et al. Clinical Features of COVID-19 Patients with Different Outcomes in Wuhan: A Retrospective Observational Study. *Biomed Res Int* 2020; **2020**: 2138387.
56. Wei JF, Huang FY, Xiong TY, et al. Acute myocardial injury is common in patients with covid-19 and impairs their prognosis. *Heart* 2020.
57. Wu Y, Hou B, Liu J, Chen Y, Zhong P. Risk Factors Associated With Long-Term Hospitalization

in Patients With COVID-19: A Single-Centered, Retrospective Study. *Front Med (Lausanne)* 2020; **7**: 315.
58. Xu PP, Tian RH, Luo S, et al. Risk factors for adverse clinical outcomes with COVID-19 in China: a multicenter, retrospective, observational study. *Theranostics* 2020; **10**(14): 6372-83.
59. Zeng JH, Wu WB, Qu JX, et al. Cardiac manifestations of COVID-19 in Shenzhen, China. *Infection* 2020.
60. Zhang G, Hu C, Luo L, et al. Clinical features and short-term outcomes of 221 patients with COVID-19 in Wuhan, China. *J Clin Virol* 2020; **127**: 104364.
61. Zhang Q, Xu Q, Chen Y, et al. Clinical characteristics of 41 patients with pneumonia due to 2019 novel coronavirus disease (COVID-19) in Jilin, China. 2020; **20**(1): 961.
62. Yang X, Yu Y, Xu J, et al. Clinical course and outcomes of critically ill patients with SARS-CoV-2 pneumonia in Wuhan, China: a single-centered, retrospective, observational study. *The Lancet Respiratory Medicine* 2020; **8**(5): 475-81.
63. Zhao M. Comparison of clinical characteristics and outcomes of patients with coronavirus disease 2019 at different ages. 2020.
64. Zhao XY, Xu XX, Yin HS, et al. Clinical characteristics of patients with 2019 coronavirus disease in a non-Wuhan area of Hubei Province, China: a retrospective study. *BMC Infect Dis* 2020; **20**(1): 311.
65. Zhou F, Yu T, Du R, et al. Clinical course and risk factors for mortality of adult inpatients with COVID-19 in Wuhan, China: a retrospective cohort study. *The Lancet* 2020; **395**(10229): 1054-62.
